# Dietary dimethylglycine sodium salt attenuates lipopolysaccharide-associated hepatic alterations and modulates redox responses in broiler chickens

**DOI:** 10.1016/j.psj.2026.107994

**Published:** 2026-10-20

**Authors:** Luyi Jiang, Yaqi Rao, Kaiwen Bai

**Affiliations:** a College of Biology and Environmental Engineering, Zhejiang Shuren University, Hangzhou, 310023, China; b School of Biological and Chemical Engineering, Zhejiang University of Science and Technology, Hangzhou, 310023, China; c Institute of Dairy Science, Ministry of Education Key Laboratory of Molecular Animal Nutrition, College of Animal Sciences, Zhejiang University, Hangzhou, 310058, China

**Keywords:** Broiler, Dimethylglycine sodium salt, Liver, Lipopolysaccharide, Meat quality

## Abstract

Repeated lipopolysaccharide (LPS) exposure compromises broiler growth and hepatic homeostasis. This study investigated the effects of dietary dimethylglycine sodium salt (DMG-Na) on hepatic responses to repeated LPS challenge and breast-muscle quality. A total of 320 one-day-old male Arbor Acres broilers were assigned to a 2 × 2 factorial design with 0 or 0.4 g DMG-Na/kg basal diet and saline or LPS (10 cages of 8 birds per treatment). Lipopolysaccharide (1 mg/kg body weight) was administered intraperitoneally on d 17, 19, and 21. DMG-Na × LPS interactions were detected for postchallenge average daily gain (*P* = 0.038), feed conversion ratio (*P* = 0.031), hepatic histopathological score (*P* = 0.013), and swollen mitochondrial profiles (*P* = 0.002). Relative to LPS alone, DMG-Na supplementation reduced histopathological score, mitochondrial swelling, and fraction-associated protein carbonyl content and was accompanied by lower hepatic SIRT1 protein abundance and phosphorylated NF-κB p65 abundance in LPSD birds (*P* < 0.05). Factorial transcriptomics and metabolomics identified 138 interaction-associated genes and 126 metabolite features, respectively, involving inflammatory and redox-related processes. Hepatic mtDNA abundance increased, whereas ATP and respiratory-chain complex activities remained unchanged. Breast-muscle drip loss showed an interaction (*P* = 0.041), but supplementation did not significantly alter drip loss within LPS-challenged birds. The treatment-only regression model outperformed the hepatic-variable random forest in predicting drip loss. Dietary DMG-Na improved postchallenge feed efficiency and attenuated hepatic inflammatory, redox, and structural alterations in LPS-challenged broilers.

## Introduction

Immune activation is a major physiological challenge to efficient broiler production because resources required for growth are redirected toward inflammatory and acute-phase responses. Lipopolysaccharide (LPS), a constituent of the outer membrane of Gram-negative bacteria, activates innate immune signaling and is widely used to investigate immune stress in poultry. Repeated LPS exposure has been associated with reduced growth performance, altered feed utilization, and changes in systemic metabolic indices (Shi et al., 2022). Nutritional approaches that modify these responses have consequently received increasing attention (Zhi et al., 2024). The liver is particularly relevant to this process because it coordinates nutrient metabolism, lipid synthesis and transport, acute-phase responses, and the handling of circulating endotoxin. Hepatic changes during immune activation can therefore contribute to metabolic disturbances accompanying impaired broiler performance (Bi et al., 2022). Hepatic responses to LPS involve closely related inflammatory, oxidative, and mitochondrial processes. Endotoxin-associated innate immune signaling engages NF-κB-dependent transcription, with the p65 subunit participating in the regulation of inflammatory mediators. Sustained inflammatory stimulation can also alter cellular oxidation-reduction balance, promote oxidative modification of proteins, and disturb mitochondrial structure. Studies in chickens have documented changes in hepatic inflammatory and redox pathways following LPS exposure (Bi et al., 2022), while mitochondrial dynamics and antioxidant signaling have been implicated in nutritional protection against LPS-associated hepatic injury (Yuan et al., 2025). Sirtuin 1 (SIRT1), an NAD^+^-dependent deacetylase involved in metabolic regulation, has functional connections with p65-dependent transcription (Yeung et al., 2004). The SIRT1-NF-κB relationship has also been examined in liver inflammation (de Gregorio et al., 2020). These findings support examining hepatic SIRT1 protein abundance and NF-κB p65 phosphorylation during LPS challenge.

Mitochondria are involved in both cellular energy metabolism and oxidative stress responses, and changes in their structure can accompany inflammatory injury. Mitochondrial swelling and disruption of cristae reflect ultrastructural alterations, whereas mitochondrial DNA abundance, ATP concentration, and respiratory-chain activities describe distinct aspects of mitochondrial status. Similarly, protein carbonyl content characterizes oxidative protein modification and complements measurements of antioxidant enzyme activity and lipid peroxidation. Liver histopathology, quantitative mitochondrial morphology, and redox measurements capture complementary aspects of injury. Hepatic transcriptomic and metabolomic profiling has also identified immune-related, amino acid, and lipid-metabolic disturbances following LPS exposure in broilers (Bi et al., 2022). Dimethylglycine (DMG) is an intermediate in the metabolism of betaine to sarcosine and glycine, linking choline-derived one-carbon metabolism with amino acid turnover. Its metabolic position has stimulated interest in dietary DMG sodium salt (DMG-Na) as a nutritional supplement. Kalmar et al. (2011) reported dose- and dietary-fat-dependent effects of DMG supplementation on broiler performance, abdominal fat deposition, and circulating metabolites. Subsequent studies in growth-restricted pigs described changes in intestinal redox status and lipid metabolism following DMG-Na supplementation (Bai et al., 2022, 2024). More recently, dietary DMG-Na improved growth performance and altered hepatic redox and mitochondrial indices in heat-stressed broilers (Bai et al., 2026). These findings establish nutritional and metabolic responses to DMG-Na under several physiological conditions. However, its effects during repeated LPS exposure, particularly on quantitative hepatic lesions, mitochondrial ultrastructure, and the accompanying inflammatory and metabolic profiles, remain insufficiently characterized in broiler chickens.

Immune-associated metabolic disturbances can extend beyond the liver to tissues of direct production value. Breast-muscle drip loss, an indicator of postmortem water-holding capacity, is influenced by glycolytic changes and the physicochemical properties of muscle proteins (Przybylski et al., 2022). Dietary interventions during oxidative stress have also been examined in relation to broiler meat quality (Zhang et al., 2022). Whether dietary DMG-Na influences postmortem muscle quality during LPS challenge, or whether hepatic characteristics are associated with drip loss independently of treatment allocation, remains unclear. The present study evaluated dietary DMG-Na supplementation at 0.4 g/kg basal diet in broilers subjected to repeated LPS challenge using a 2 × 2 factorial design. Growth performance, circulating biochemical indices, hepatic histopathology, mitochondrial ultrastructure, redox-related endpoints, mitochondrial DNA abundance, and respiratory-chain measurements were assessed. Hepatic SIRT1 protein abundance and NF-κB p65 phosphorylation were examined together with selected transcript measurements. Factorial transcriptomic and metabolomic analyses were used to characterize diet-dependent differences in the hepatic response to LPS. Breast-muscle quality and treatment-adjusted associations between hepatic variables and drip loss were also investigated. We hypothesized that dietary DMG-Na would attenuate LPS-associated impairments in growth and hepatic structural integrity, accompanied by changes in hepatic inflammatory and redox-related responses.

## Materials and methods

### Animal ethics

All animal procedures were approved by the Animal Care and Use Committee of Zhejiang University of Science and Technology, Hangzhou, China (approval no. 20260171), and conducted under the approved animal-care protocol.

### Experimental design, birds, and diets

A total of 320 one-day-old male Arbor Acres broilers of similar initial weight were randomly allocated to a 2 × 2 factorial design: basal diet plus saline (CON), DMG-Na diet plus saline (COND), basal diet plus LPS (LPS), and DMG-Na diet plus LPS (LPSD). Each treatment comprised 10 cages of eight birds. Dietary DMG-Na and LPS exposure were the experimental factors. DMG-Na (99.9% purity; Qilu Sheng Hua Pharmaceutical Co., Ltd., Shandong, China) was added at 0.4 g/kg basal feed, without ingredient replacement. The 400 mg/kg inclusion level was previously evaluated in heat-stressed broilers (Bai et al., 2026) and falls within the 0.1∼1.0 g/kg range investigated by Kalmar et al. (2011).

On d 17, 19, and 21, LPS and LPSD birds received intraperitoneal *E. coli* O55:B5 LPS (1 mg/kg body weight; Sigma-Aldrich, St. Louis, MO, USA; L2880) freshly prepared in sterile 0.9% saline. CON and COND birds received an equivalent volume of saline. The basal diet was formulated to meet Arbor Acres nutrient recommendations (Table S1). DMG-Na was added to the unchanged basal formulation. Feed and water were provided ad libitum. The brooding temperature was gradually reduced from approximately 34°C during the first week to 26°C on day 21. Continuous lighting was maintained, and the birds were inspected daily.

### Growth performance

Birds and residual feed were weighed by cage on d 1, 16, and 21. Average daily gain (ADG), average daily feed intake (ADFI), and feed conversion ratio (FCR, feed intake/weight gain) were calculated for d 1∼15 and d 16∼21, with cage as the experimental unit.

### Sample collection

On d 21, 3 h after the final injection, one bird nearest the cage mean weight was selected per cage (n = 10 per treatment). Wing-vein blood was collected into serum tubes before cervical dislocation. After 30 min of clotting, serum was separated at 3,000 × g for 10 min at 4°C and stored at −80°C. Liver tissue was divided for histology, transmission electron microscopy (TEM), biochemical assays, mitochondrial enrichment, RNA sequencing, qPCR, and metabolomics. Molecular samples were snap-frozen and stored at −80°C. Left pectoralis major muscle was collected for meat quality measurements.

### Hepatic histology and ultrastructural examination

Liver samples (one bird per cage) were fixed in 4% paraformaldehyde, paraffin-embedded, sectioned at 5 μm, and stained with hematoxylin and eosin. Two nonconsecutive sections with five nonoverlapping fields each were examined per bird. Two treatment-blinded observers scored each bird independently and resolved discrepancies by consensus. Lobular inflammation and portal inflammation were each scored from 0 (absent) to 3 (severe). Necrosis was scored 0 (absent), 1 (< 10% parenchyma), 2 (10%∼25%), or 3 (> 25%). Component scores were summed to a bird level histopathological score of 0∼9, with higher scores indicating more extensive lesions.

For TEM, approximately 1 mm³ liver specimens were fixed in 2.5% glutaraldehyde, postfixed in 1% osmium tetroxide, epoxy-embedded, sectioned, and stained with uranyl acetate and lead citrate. Five nonoverlapping micrographs at a consistent calibrated magnification and at least 100 evaluable mitochondrial profiles were assessed per bird. Profiles with substantial sectioning artifacts or indistinct boundaries were excluded. Mitochondrial swelling was defined by an enlarged or rounded profile with matrix rarefaction and reduced electron density. Abnormal cristae were identified by fragmented, disorganized, or markedly reduced internal membranes. Blinded assessments yielded percentages of swollen profiles and profiles with abnormal cristae per bird. Fields and organelle profiles were subsamples. Each bird contributed one value per endpoint.

### Serum lipid and glucose measurements

Serum total cholesterol (TC), triglycerides (TG), and glucose were determined using cholesterol oxidase-peroxidase, glycerol phosphate oxidase-peroxidase, and glucose oxidase methods, respectively. Low- and high-density lipoprotein cholesterol (LDL-C and HDL-C) were measured by selective enzymatic colorimetry. Commercial kits (Nanjing Jiancheng Bioengineering Institute, Nanjing, China) were used with kit-specific calibrators and blanks. Absorbance was read by microplate reader.

### Serum enzyme activity measurements

Serum alanine aminotransferase (ALT) and aspartate aminotransferase (AST) activities were determined colorimetrically by Reitman-Frankel reactions, alkaline phosphatase (ALP) by disodium phenyl phosphate hydrolysis, and lactate dehydrogenase (LDH) by NADH oxidation. Commercial kits (Nanjing Jiancheng Bioengineering Institute) were used according to the manufacturer's calibration, blank-correction, and reaction-time procedures.

### Serum and hepatic redox status

Total antioxidant capacity (T-AOC), superoxide dismutase (SOD), glutathione peroxidase (GSH-Px), and malondialdehyde (MDA) were measured in serum and liver homogenates using ferric reducing capacity, xanthine oxidase inhibition, glutathione-dependent substrate conversion, and thiobarbituric-acid reactions, respectively. Liver was homogenized at 10% (w/v) in chilled saline and centrifuged at 4°C. Commercial kits (Nanjing Jiancheng Bioengineering Institute) were used. Hepatic values were normalized to protein measured by the Coomassie brilliant blue assay.

### Redox indices in hepatic mitochondria-enriched fractions

Hepatic mitochondria-enriched fractions were prepared by differential centrifugation using a commercial isolation kit. MnSOD activity was measured by inhibition of superoxide-dependent chromogen formation, GSH-Px by a glutathione-coupled reaction, and γ-glutamylcysteine ligase (γ-GCL) by enzymatic colorimetry. Protein carbonyls were measured after 2,4-dinitrophenylhydrazine derivatization and 8‑hydroxy-2′-deoxyguanosine (8-OHdG) by ELISA (Nanjing Jiancheng Bioengineering Institute). Fraction-associated values were normalized to protein concentration.

### Hepatic ATP, mtDNA abundance, and respiratory-chain enzyme activities

Hepatic ATP was quantified using a luciferin-luciferase assay. Relative mitochondrial DNA (mtDNA) abundance was measured by qPCR of the mitochondrial d-loop (forward, 5′-AGGACTACGGCTTGAAAAGC-3′; reverse, 5′-CATCTTGGCATCTTCAGTGCC-3′; 198 bp), normalized to the β-actin locus (forward, 5′-TTCTTGGGTATGGAGTCCTG-3′; reverse, 5′-TAGAAGCATTTGCGGTGG-3′; 150 bp). Respiratory-chain complex I∼V activities were assayed in mitochondria-enriched fractions using commercial kits: NADH oxidation (I), 2,6-dichlorophenolindophenol reduction (II), cytochrome c reduction (III), reduced cytochrome c oxidation (IV), and ATP hydrolysis (V). Complex activities were normalized to fraction protein.

### Liver transcriptome sequencing and bioinformatic analysis

Ten liver samples per treatment (one bird per cage, n = 40) were subjected to RNA sequencing. Total RNA was extracted using TRIzol. RNA purity and concentration were assessed spectrophotometrically, and integrity was determined using an Agilent 2100 Bioanalyzer. Samples with RNA integrity number > 7.0 were used to prepare poly(A)-enriched, stranded libraries (TruSeq Stranded mRNA Library Prep Kit; Illumina) and sequenced on a NovaSeq 6000 (paired-end, 2 × 150 bp). Reads were trimmed and quality-filtered with fastp v0.23.4 (Chen et al., 2018). Libraries yielded 40.7∼64.7 million clean reads (6.11∼9.63 Gb), with Q30 of 94.18%∼95.11%. Sequencing reads were aligned to the Gallus gallus reference genome bGalGal1.mat.broiler.GRCg7b (GCF_016699485.2, annotation release 106) using HISAT2 v2.2.1 (Kim et al., 2019). The total and unique mapping rates ranged from 93.56% to 95.25% and approximately 88% to 91%, respectively (Table S2).

Gene-level counts were obtained using featureCounts v2.0.1 (Liao et al., 2014) and modeled in DESeq2 v1.40.2 (Love et al., 2014) with the design ∼ DMG + LPS + DMG:LPS. The primary contrast was the DMG-Na × LPS interaction, (LPSD - LPS) - (COND - CON), at Benjamini-Hochberg-adjusted *P* < 0.05. Secondary comparisons included COND versus CON, LPS versus CON, LPSD versus LPS, and LPSD versus COND. An additional threshold of |log₂ fold change| ≥ 2 was applied to identify differentially expressed genes (DEGs) in pairwise comparisons. Variance-stabilized counts were used for principal-component analysis, and interaction-associated genes underwent Gene Ontology and KEGG enrichment analyses, including ranking by the signed interaction statistic (Tables S3 and S4A-B).

### Untargeted hepatic metabolomics

Ten independent liver samples per treatment (n = 40) were analyzed by untargeted metabolomics. Approximately 50 mg of frozen tissue was extracted with 400 μL chilled methanol/water (4:1, v/v) containing 0.02 mg/mL l-2-chlorophenylalanine, homogenized, sonicated for 30 min, incubated at −20°C for 30 min, and centrifuged at 13,000 × g for 15 min at 4°C. Supernatants were analyzed in separate positive- and negative-ion modes using UHPLC coupled to a Q Exactive HF-X mass spectrometer (Thermo Fisher Scientific) and an ACQUITY UPLC HSS T3 column (100 × 2.1 mm, 1.8 μm; Waters).

Pooled quality-control (QC) samples containing equal extract aliquots were injected after every eight biological samples. Injection order was randomized within each ionization-mode batch. Progenesis QI v3.0 was used for feature extraction, alignment, and peak integration. Features detected in < 80% of biological samples were removed. Run-order drift was corrected by pooled-QC-based robust LOESS when present (Broadhurst et al., 2018), after which features with pooled-QC relative standard deviation >30% were removed. Remaining missing values were imputed by k-nearest neighbors (Do et al., 2018), followed by log₂ transformation. QC filtering and correction were evaluated separately by ionization mode (Tables S5 and S6A-B).

Factorial feature-level analysis used limma empirical-Bayes models (Ritchie et al., 2015), with the primary DMG-Na × LPS interaction defined as (LPSD - LPS) - (COND - CON) and Benjamini-Hochberg-adjusted *P* < 0.05. Four secondary simple-effect contrasts were assessed. Pairwise differential features also required fold change > 2 or < 0.5. OPLS-DA and variable importance in projection (VIP) were exploratory, with validation statistics in Table S7C (Triba et al., 2015). Metabolites were annotated by accurate mass, retention behavior, and MS/MS library evidence using Metabolomics Standards Initiative confidence levels (Sumner et al., 2007; Table S8). KEGG pathway analyses were performed in MetaboAnalyst 6.0 (Pang et al., 2024). Model coefficients for DMG-Na and LPS represent COND versus CON and LPS versus CON under the CON reference condition, rather than marginal average effects (Tables S9A-B).

### Quantitative real-time PCR

Total RNA was extracted from liver tissues using TRIzol reagent, and RNA concentration and purity were assessed spectrophotometrically. Subsequently, 1 μg of total RNA was reverse-transcribed into cDNA in a 20 μL reaction at 37°C for 15 min, followed by 85°C for 5 s. Quantitative real-time PCR was performed using SYBR Green chemistry on a QuantStudio 5 system (Applied Biosystems). Each 20 μL reaction contained 10 μL of 2 × SYBR Green Master Mix, 0.4 μL of each primer (10 μM), 2 μL of cDNA, and 7.2 μL of nuclease-free water. Amplification consisted of 95°C for 2 min, followed by 40 cycles of 95°C for 15 s and 60°C for 60 s. Melting-curve analysis was performed to confirm amplification specificity. Primer sequences and GenBank accession numbers are listed in Table S10. Relative gene expression was calculated using the $2^{-\Delta \Delta C_{t}}$ method with *β-actin* as the reference gene and CON as the calibrator group (Livak and Schmittgen, 2001).

### Hepatic SIRT1 and NF-κB p65 protein analyses

Total hepatic proteins were extracted from liver tissue using lysis buffer containing protease and phosphatase inhibitors. Protein concentrations were determined by bicinchoninic acid assay. Equal amounts of protein were separated by SDS-PAGE and transferred to polyvinylidene difluoride membranes. Membranes were blocked in 5% (w/v) bovine serum albumin in Tris-buffered saline containing 0.1% Tween 20 (TBST) for 1 h at room temperature. Primary antibodies against SIRT1 (#2028), total NF-κB p65 (#8242), phosphorylated NF-κB p65 (Ser536; #3033), and β-actin (#4970) (Cell Signaling Technology, Danvers, MA, USA) were diluted 1:1,000 and incubated overnight at 4°C. After three 5-min washes in TBST, membranes were incubated for 1 h at room temperature with the corresponding horseradish peroxidase-conjugated secondary antibodies (Cell Signaling Technology, 1:2,000), washed three times in TBST, and developed using enhanced chemiluminescence. Band intensities were quantified by densitometry. SIRT1, total NF-κB p65, and phosphorylated NF-κB p65 (Ser536) were normalized to their corresponding β-actin loading controls. The phosphorylated-to-total p65 ratio was calculated for each biological sample using the matched β-actin-normalized signals. Normalized protein abundances and phosphorylation ratios were expressed relative to the corresponding CON group mean.

### Meat quality measurements

The left pectoralis major muscle from one bird per cage (n = 10 per treatment) was sampled from standardized cranial-central regions. Separate portions were allocated to pH, drip loss, cooking loss, shear force, and color measurements. Muscle pH was measured at 1 and 24 h postmortem using a penetration pH meter (Testo 205; Testo, Lenzkirch, Germany) calibrated with pH 4.01 and 7.00 buffers. Two readings taken 1∼2 cm deep at adjacent unpunctured sites were averaged per bird. Samples were held at 4°C before the 24 h measurement. Drip loss was measured by the suspension method of Honikel (1998). Approximately 15 g of pectoralis major (3.0 × 2.0 × 2.5 cm) was weighed (ME204 balance, Mettler-Toledo), suspended without contact with the container or exudate, and held at 4°C for 24 h. After blotting, samples were reweighed. Drip loss (%) = [(initial - final weight)/initial weight] × 100. For cooking loss, approximately 30 g of adjacent muscle was weighed, vacuum-sealed, and heated in an 80°C water bath until the core reached 75°C (Testo 108 thermometer). Samples were cooled to approximately 20°C, blotted, and reweighed. Cooking loss (%) = [(raw - cooked weight)/raw weight] × 100.

Warner-Bratzler shear force was measured using a TA.XTplus texture analyzer (Stable Micro Systems) with a V-shaped blade. Three cooked strips (1.0 × 1.0 × 3.0 cm) were sheared perpendicular to the muscle fibers at pretest, test, and posttest speeds of 2.0, 1.0, and 10.0 mm/s, respectively. Peak force (N) was averaged across strips per bird. At 24 h postmortem, CIE L*, a*, and b* values were measured using a CR-400 chroma meter (Konica Minolta; D65 illuminant, 8-mm aperture), calibrated with the supplied white plate. After a 30 min bloom at 20°C, three readings were obtained from nonoverlapping muscle sites free of visible discoloration or moisture and averaged per bird.

### Association and random-forest regression analyses

Matched hepatic and drip-loss data were available for 40 birds (10 per treatment). Separate linear models related continuous drip loss to eight prespecified variables: SIRT1, NF-κB p65, NLRP3, fraction-associated protein carbonyls, mtDNA abundance, NDUFA2, GSH-Px activity, and serum ALT activity. Each model included DMG-Na, LPS, their interaction, and one SD-standardized predictor. Predictor-specific *P* values were adjusted by Benjamini-Hochberg across eight tests. Pooled and within-treatment Spearman correlations were also calculated (Tables S11A-B). Regression coefficients are expressed as percentage-point differences in drip loss per one-SD increase in each predictor.

Random-forest regression used five treatment-stratified outer folds repeated 10 times and four inner folds for tuning. Each outer test fold contained two birds per treatment. Predictor scaling, near-zero-variance and correlation filtering (|r| > 0.80), and tuning were restricted to training folds. Models contained 1,000 trees, with mtry and minimum terminal-node size selected by inner cross-validation. Treatment assignment determined stratification but was excluded from hepatic predictors (Varma and Simon, 2006; Vabalas et al., 2019). Out-of-fold predictions were evaluated by root mean square error (RMSE), mean absolute error (MAE), and R². Ninety-five percent confidence intervals were estimated by 2,000 bird-level bootstrap resamples. The hepatic model was compared with an outer-training mean (null) and a treatment-only linear model (DMG-Na, LPS, and interaction), using identical outer partitions. Permutation importance was summarized across resamples. Analyses used R v4.5.0 and ranger, rsample, recipes, tune, yardstick, and boot.

### Statistical analysis

Phenotypic data were analyzed in SAS 9.4 (SAS Institute Inc., Cary, NC, USA) using two-way analysis of variance for a completely randomized 2 × 2 factorial design. Dietary DMG-Na (0 or 0.4 g/kg basal diet), LPS challenge (saline or LPS), and their interaction were included as fixed effects. The model was Yᵢⱼₖ = μ + Dᵢ + Lⱼ + (D × L)ᵢⱼ + εᵢⱼₖ, where Dᵢ and Lⱼ denote the dietary and challenge effects, respectively, and εᵢⱼₖ represents the residual for replicate cage k. The cage was the experimental unit for growth performance (10 cages per treatment). For serum, hepatic, and breast-muscle outcomes, one sampled bird represented each independent cage (n = 10 per treatment). Technical measurements and microscopic subsamples were averaged to obtain a single observation for each endpoint per bird. Mitochondrial-profile proportions were calculated separately for each bird before statistical analysis.

Model residuals were assessed for normality and homogeneity of variance. When the DMG-Na × LPS interaction was significant, treatment means were compared using Tukey-adjusted pairwise tests. The LPSD versus LPS pairwise contrast was evaluated independently of the factorial interaction test. In the absence of a significant interaction, the main effects of diet and LPS were evaluated. Phenotypic data are presented as mean ± SD, and *P* < 0.05 was considered significant.

## Results

### Growth performance

Before the LPS challenge (d 1∼15), ADG, ADFI, and feed conversion ratio (FCR) were comparable among treatments (*P* > 0.05; Fig. 1). During d 16∼21, ADG showed significant effects of dietary DMG-Na (*P*_D_ = 0.019), LPS (*P*_L_< 0.001), and their interaction (*P*_D×L_ = 0.038). ADG was greater in LPSD than LPS (*P* < 0.05), although both challenged groups had lower ADG than the saline-treated groups. FCR also showed significant diet (*P*_D_ = 0.006), LPS (*P*_L_ < 0.001), and interaction effects (*P*_D×L_ = 0.031), with a lower FCR in LPSD than LPS (*P* < 0.05). ADFI did not differ significantly among treatments (*P* > 0.05).

**Fig. 1:**
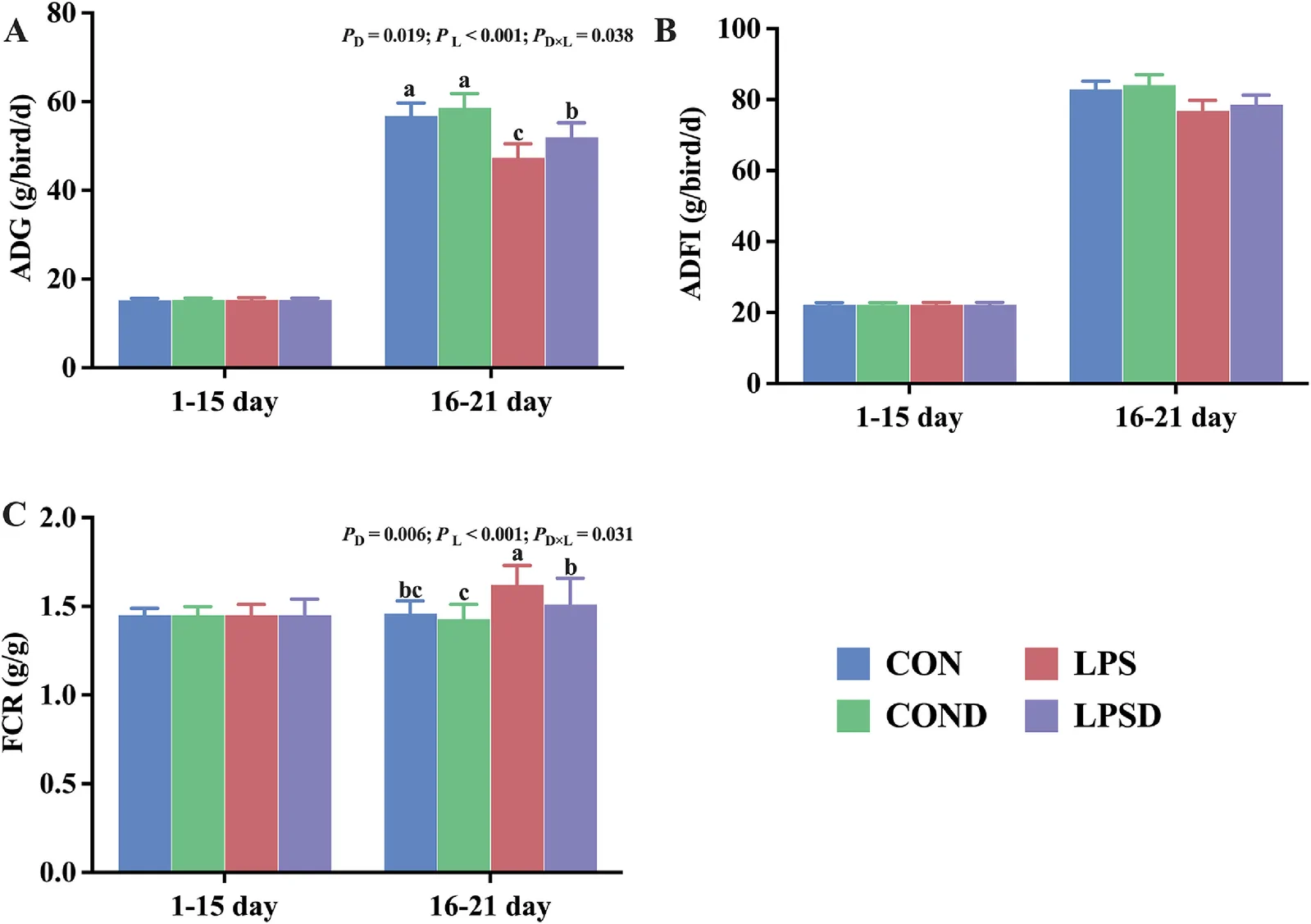
Effects of dietary dimethylglycine sodium salt (DMG-Na) and lipopolysaccharide (LPS) challenge on broiler growth performance. (A) Average daily gain (ADG), (B) average daily feed intake (ADFI), and (C) feed conversion ratio (FCR) during d 1∼15 and d 16∼21. CON, basal diet + saline; COND, DMG-Na-supplemented diet + saline; LPS, basal diet + LPS; LPSD, DMG-Na-supplemented diet + LPS. Data are means ± SD (10 independent cages per treatment, eight birds per cage). *P*_D_, dietary DMG-Na main effect; *P*_L_, LPS main effect; *P*_D×L_, diet × LPS interaction. Different lowercase letters indicate treatment means differing at *P* < 0.05.

### Hepatic histology and ultrastructure

Histological examination revealed regular hepatic architecture in CON and COND birds, whereas LPS-challenged birds exhibited hepatocellular alterations and inflammatory lesions (Fig. 2A). These lesions were less extensive in LPSD birds. Significant main effects of diet (*P*_D_ < 0.001) and LPS (*P*_L_ < 0.001), as well as a significant diet × LPS interaction (*P*_D×L_ = 0.013), were observed for histopathological scores (Fig. 2C). Compared with LPS, LPSD had a lower lesion score (*P* < 0.05). The LPSD score remained higher than those of the saline-treated groups (*P* < 0.05), whereas CON and COND did not differ (*P* > 0.05).

**Fig. 2:**
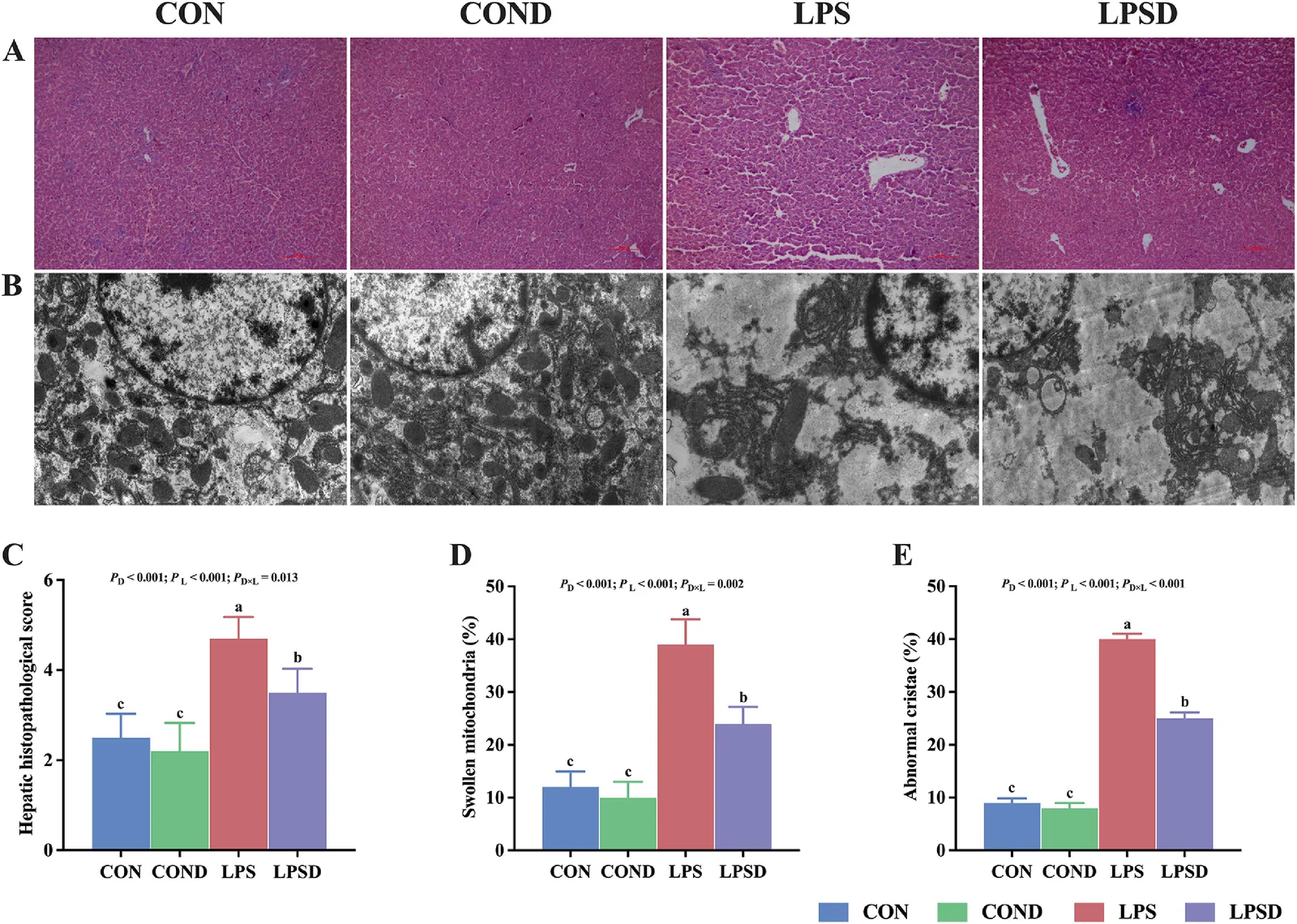
Hepatic histological lesions and mitochondrial ultrastructure of broilers given dietary dimethylglycine sodium salt (DMG-Na) and challenged with lipopolysaccharide (LPS). (A) Representative hematoxylin-and-eosin-stained liver sections; (B) representative transmission electron micrographs; (C) hepatic histopathological score (range 0-9); (D) swollen mitochondrial profiles (%); and (E) mitochondrial profiles with abnormal cristae (%). Scale bars are 100 μm in A and 1 μm in B. CON, basal diet + saline; COND, DMG-Na-supplemented diet + saline; LPS, basal diet + LPS; LPSD, DMG-Na-supplemented diet + LPS. Values are means ± SD (n = 10 birds per treatment, one bird from each independent replicate cage). *P*_D_, dietary DMG-Na main effect; *P*_L_, LPS main effect; *P*_D×L_, interaction. Different lowercase letters indicate *P* < 0.05.

Transmission electron microscopy showed enlarged mitochondrial profiles, reduced matrix electron density, and disorganized cristae in the LPS group, with fewer abnormalities in LPSD (Fig. 2B). The proportion of swollen mitochondrial profiles was significantly affected by diet (*P*_D_ < 0.001), LPS challenge (*P*_L_ < 0.001), and their interaction (*P*_D×L_ = 0.002; Fig. 2D). A significant difference was observed between the LPSD and LPS groups (*P* < 0.001). Similarly, diet, LPS challenge, and their interaction significantly affected the proportion of mitochondrial profiles with abnormal cristae (all *P* < 0.001). This proportion was significantly lower in the LPSD group than in the LPS group (*P* < 0.001).

### Serum lipid and glucose profile

Serum TC concentrations were affected by dietary DMG-Na (*P*_D_ < 0.001), LPS (*P*_L_ < 0.001), and their interaction (*P*_D×L_ = 0.031; Fig. 3A). Corresponding effects were observed for serum TG (*P*_D_ = 0.002, *P*_L_ < 0.001, *P*_D×L_ = 0.048; Fig. 3C). Both TC and TG were elevated in LPS compared with the saline-treated groups and were lower in LPSD than in LPS (*P* < 0.05). Serum glucose, low-density lipoprotein cholesterol, and high-density lipoprotein cholesterol did not differ significantly among treatments (*P* > 0.05; Fig. 3B, D, E).

**Fig. 3:**
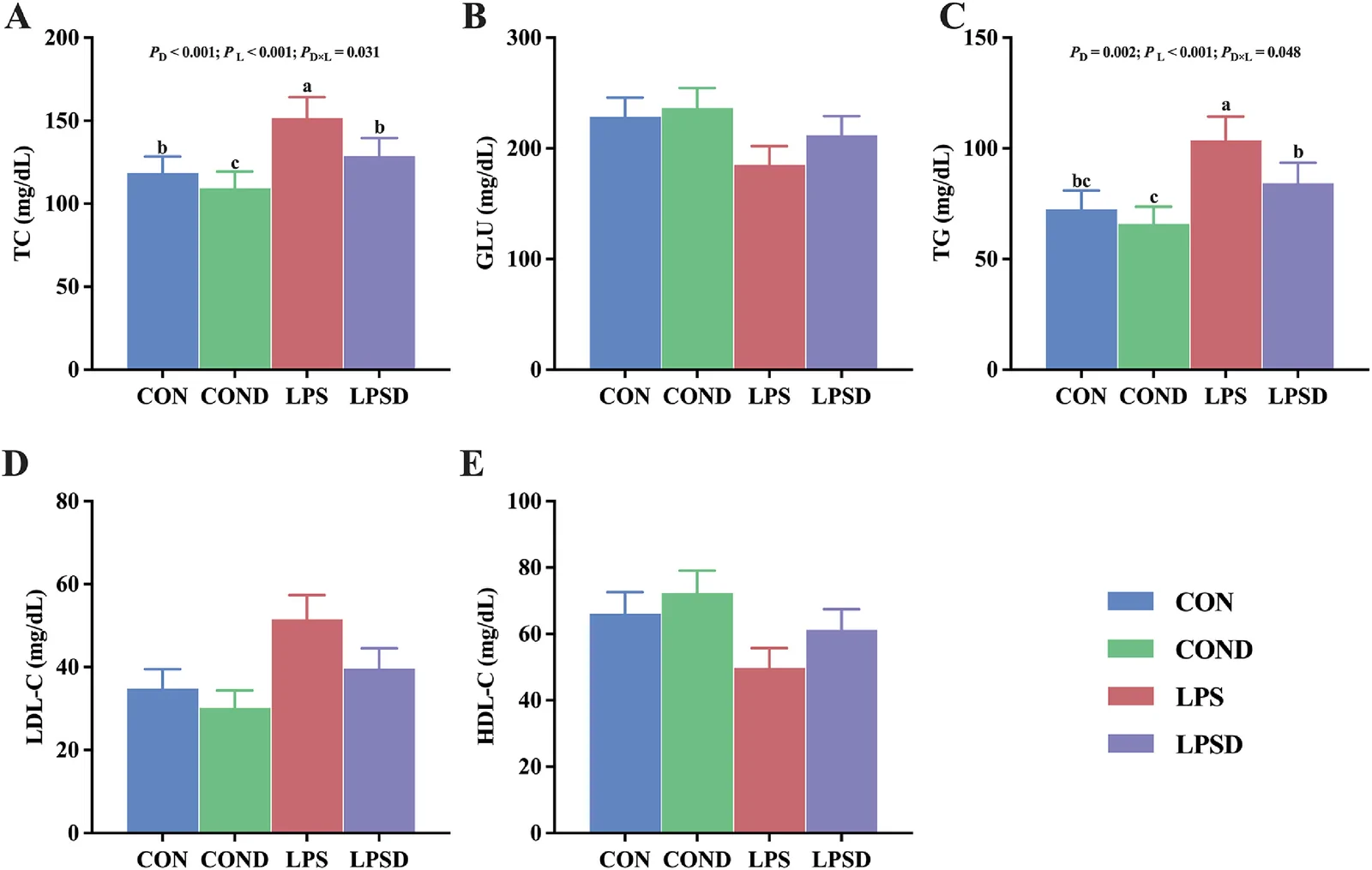
Effects of dietary dimethylglycine sodium salt (DMG-Na) and lipopolysaccharide (LPS) challenge on serum lipid and glucose concentrations in broiler chickens. (A) Total cholesterol (TC); (B) glucose (GLU); (C) triglycerides (TG); (D) low-density lipoprotein cholesterol (LDL-C); and (E) high-density lipoprotein cholesterol (HDL-C). CON, basal diet + saline; COND, DMG-Na-supplemented diet + saline; LPS, basal diet + LPS; LPSD, DMG-Na-supplemented diet + LPS. Values are means ± SD (n = 10 birds per treatment, one bird per independent replicate cage). *P*_D_, DMG-Na main effect; *P*_L_, LPS main effect; *P*_D×L_, interaction. Different lowercase letters denote differences at *P* < 0.05.

### Serum enzyme activities

Serum ALT, AST, ALP, and LDH activities showed significant DMG-Na × LPS interactions (*P*_D×L_ = 0.018, 0.021, 0.045, and 0.006, respectively; Fig. 4A-D). Dietary DMG-Na main effects were also detected for ALT (*P*_D_ = 0.002), AST (*P*_D_ = 0.003), ALP (*P*_D_ = 0.004), and LDH (*P*_D_ < 0.001), together with LPS main effects for all four enzymes (*P*_L_ < 0.001). Enzyme activities were higher in LPS than in the saline-treated groups and lower in LPSD than in LPS (*P* < 0.05).

**Fig. 4:**
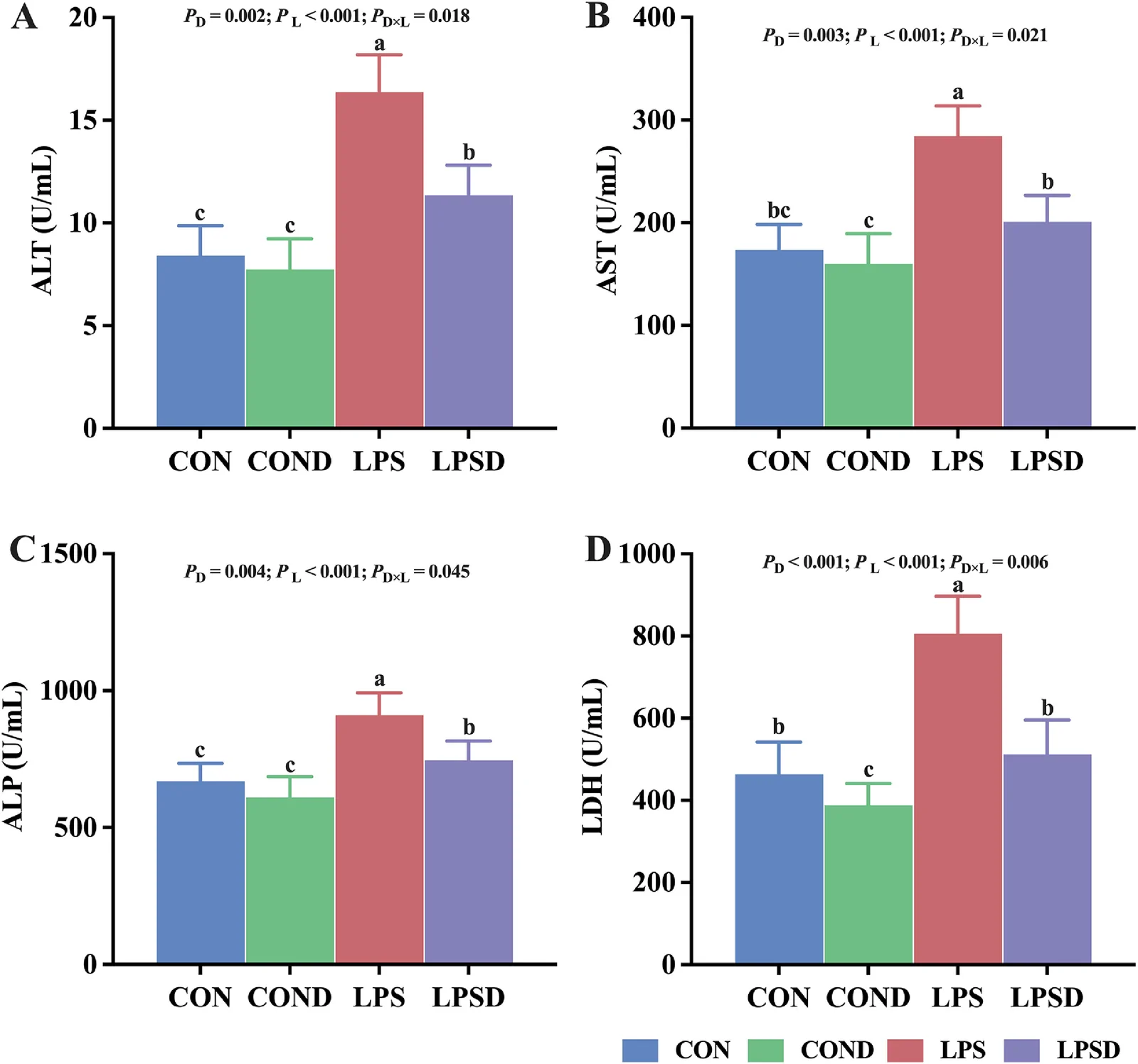
Serum enzyme activities in broilers receiving dietary dimethylglycine sodium salt (DMG-Na) and challenged with lipopolysaccharide (LPS). (A) Alanine aminotransferase (ALT), (B) aspartate aminotransferase (AST), (C) alkaline phosphatase (ALP), and (D) lactate dehydrogenase (LDH). CON, basal diet + saline; COND, DMG-Na-supplemented diet + saline; LPS, basal diet + LPS; LPSD, DMG-Na-supplemented diet + LPS. Values are means ± SD (n = 10, one bird from each independent replicate cage). *P*_D_, DMG-Na main effect; *P*_L_, LPS main effect; *P*_D×L_, interaction. Different lowercase letters indicate *P* < 0.05.

### Systemic, whole-liver, and mitochondria-enriched fraction redox indices

Serum superoxide dismutase (SOD) activity exhibited significant diet (*P*_D_ < 0.001), LPS (*P*_L_ < 0.001), and interaction effects (*P*_D×L_ = 0.032; Fig. 5A). Activity was highest in COND and lowest in LPS, with an intermediate value in LPSD. Serum total antioxidant capacity (T-AOC), glutathione peroxidase (GSH-Px) activity, and malondialdehyde (MDA) concentration showed no significant effects (*P* > 0.05; Fig. 5B-D). Likewise, SOD, T-AOC, GSH-Px, and MDA measured in whole-liver homogenates did not differ among treatments (*P* > 0.05; Fig. 5E-H).

**Fig. 5:**
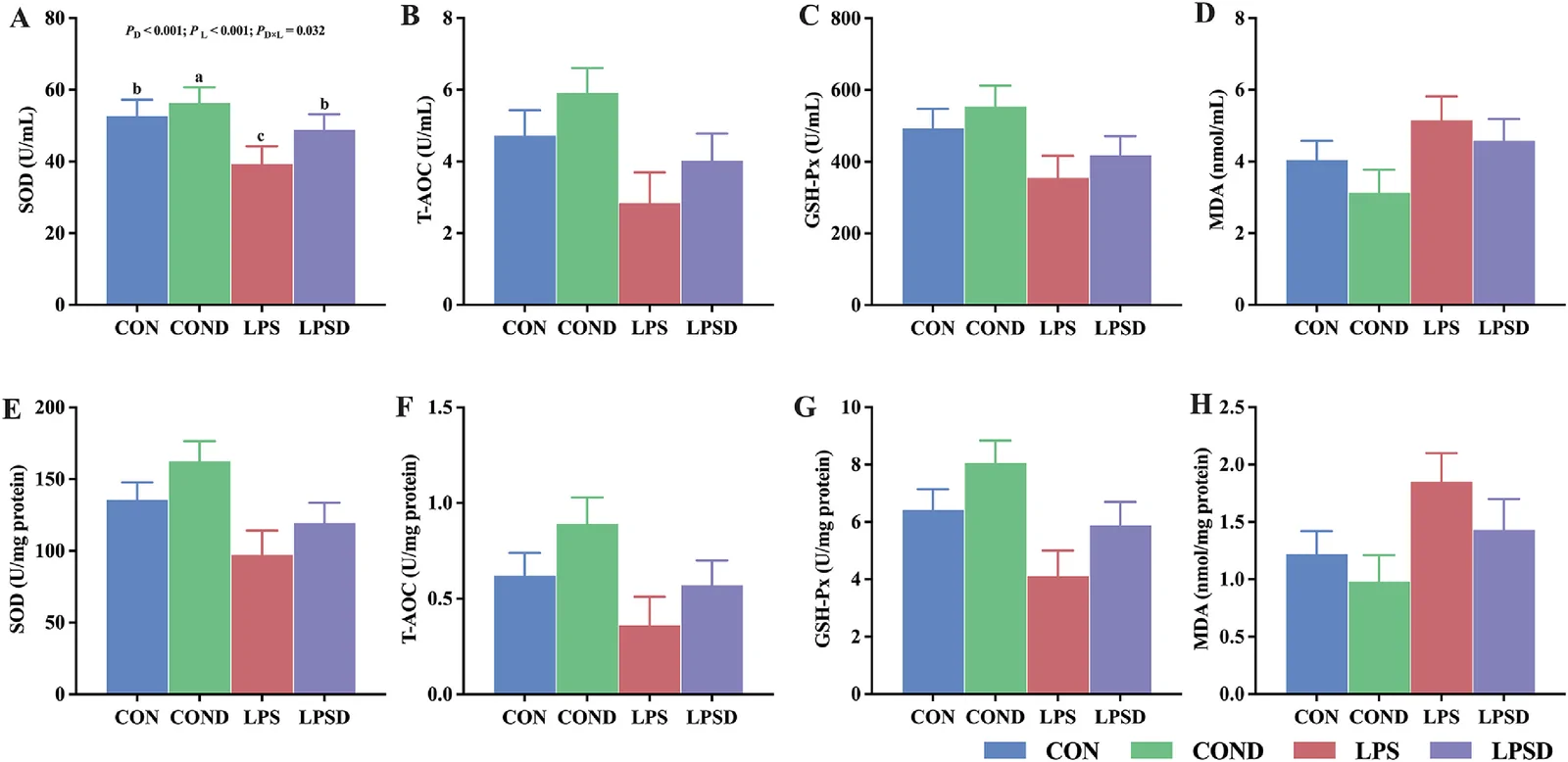
Serum and whole-liver redox-related indices in broilers receiving dietary dimethylglycine sodium salt (DMG-Na) and challenged with lipopolysaccharide (LPS). Serum: (A) superoxide dismutase (SOD) activity, (B) total antioxidant capacity (T-AOC), (C) glutathione peroxidase (GSH-Px) activity, and (D) malondialdehyde (MDA). Liver: (E) SOD, (F) T-AOC, (G) GSH-Px, and (H) MDA. CON, basal diet + saline; COND, DMG-Na-supplemented diet + saline; LPS, basal diet + LPS; LPSD, DMG-Na-supplemented diet + LPS. Values are means ± SD (n = 10, one bird per independent replicate cage). *P*_D_, main effect of DMG-Na; *P*_L_, main effect of LPS; *P*_D×L_, their interaction. Different lowercase letters indicate *P* < 0.05.

In hepatic mitochondria-enriched fractions, manganese SOD (MnSOD) activity showed effects of dietary DMG-Na (*P*_D_ < 0.001), LPS (*P*_L_ = 0.044), and their interaction (*P*_D×L_ = 0.041; Fig. 6A). Activity was greater in COND than CON (*P* < 0.05), whereas LPS and LPSD did not differ significantly. Protein carbonyl content also showed diet (*P*_D_ < 0.001), LPS (*P*_L_ < 0.001), and interaction effects (*P*_D×L_ = 0.047; Fig. 6D) and was lower in LPSD than LPS (*P* < 0.05). GSH-Px and γ-glutamylcysteine ligase activities and 8‑hydroxy-2′-deoxyguanosine concentrations in these fractions were comparable among treatments (*P* > 0.05; Fig. 6B,C,E).

**Fig. 6:**
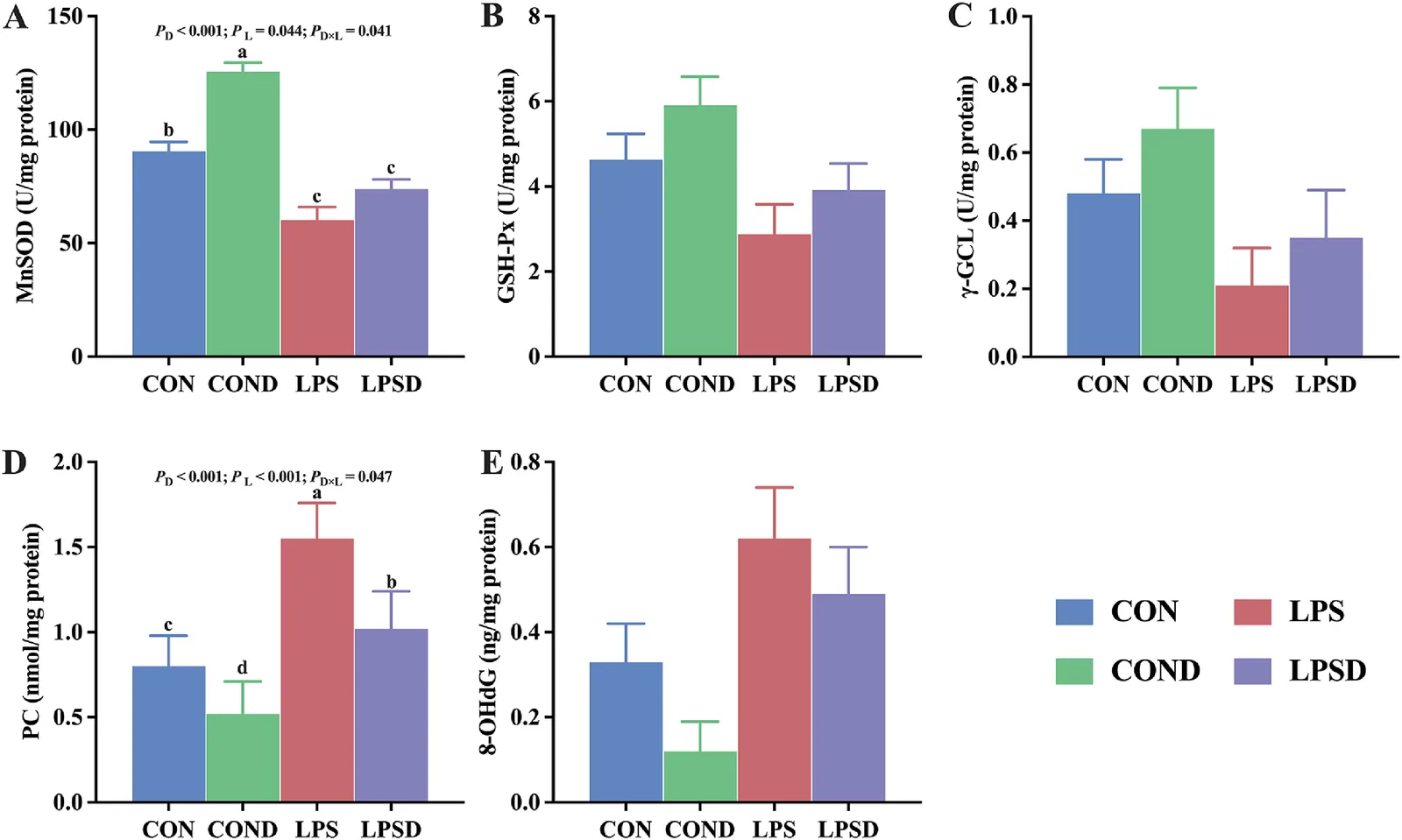
Redox-related measurements in hepatic mitochondria-enriched fractions from broilers given dietary dimethylglycine sodium salt (DMG-Na) and challenged with lipopolysaccharide (LPS). (A) Manganese superoxide dismutase (MnSOD), (B) glutathione peroxidase (GSH-Px), (C) γ-glutamylcysteine ligase (γ-GCL), (D) protein carbonyl (PC) concentration, and (E) 8‑hydroxy-2′-deoxyguanosine (8-OHdG). CON, basal diet + saline; COND, DMG-Na-supplemented diet + saline; LPS, basal diet + LPS; LPSD, DMG-Na-supplemented diet + LPS. Values are means ± SD (n = 10 birds per treatment). *P*_D_, DMG-Na main effect; *P*_L_, LPS main effect; *P*_D×L_, interaction. Different lowercase letters indicate *P* < 0.05.

### Hepatic mtDNA abundance, ATP concentration, and respiratory-chain enzyme activities

Relative hepatic mtDNA abundance was affected by dietary DMG-Na (*P*_D_ < 0.001), LPS (*P*_L_ < 0.001), and their interaction (*P*_D×L_ = 0.036; Fig. 7B). Abundance was higher in COND than CON, lowest in LPS, and higher in LPSD than LPS (*P* < 0.05). Hepatic ATP concentration and the activities of respiratory-chain complexes I-V in mitochondria-enriched fractions did not differ significantly among treatments (*P* > 0.05; Fig. 7A, C-G).

**Fig. 7:**
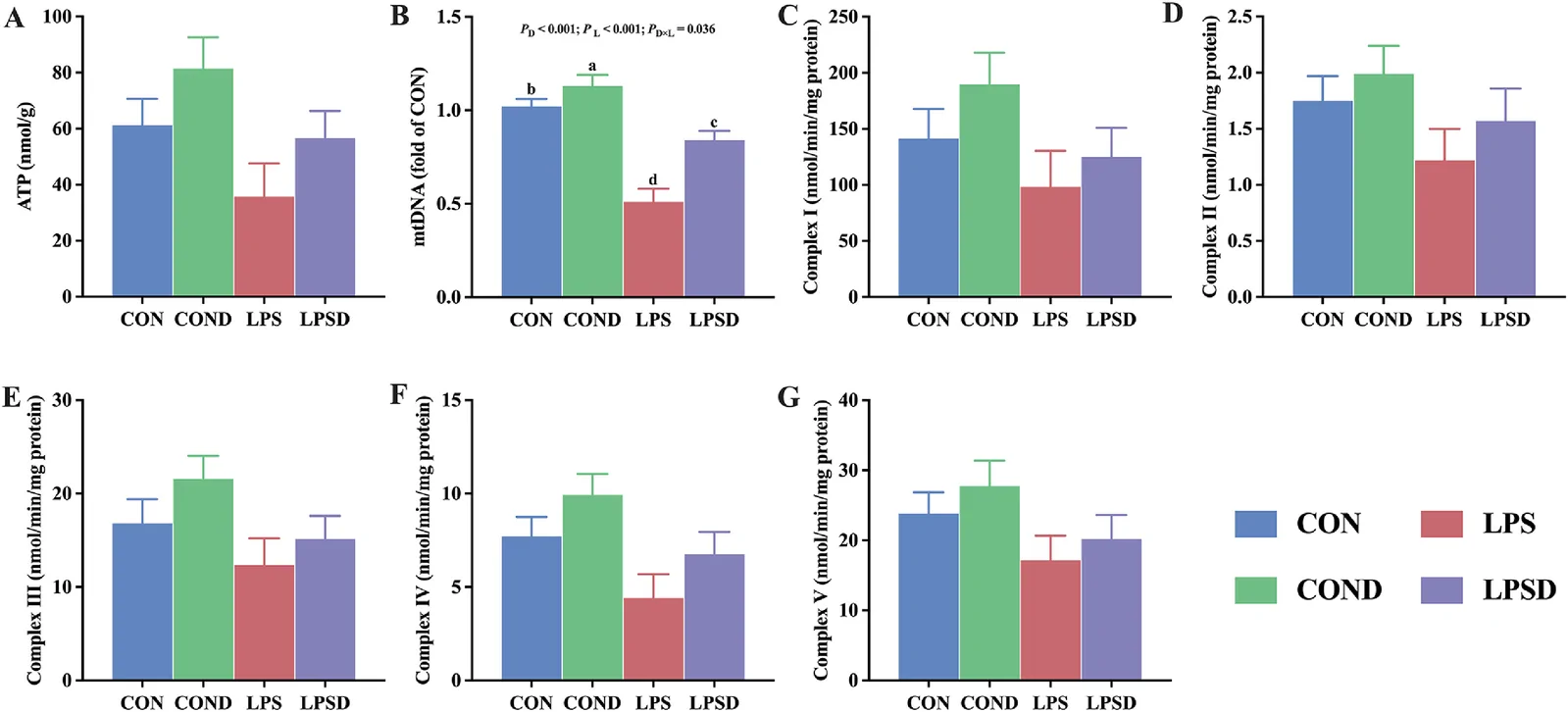
Hepatic ATP, mitochondrial DNA, and respiratory-chain enzyme measurements in broilers receiving dietary dimethylglycine sodium salt (DMG-Na) and challenged with lipopolysaccharide (LPS). (A) ATP concentration; (B) relative mitochondrial DNA (mtDNA) abundance; and activities of (C) complex I, (D) complex II, (E) complex III, (F) complex IV, and (G) complex V. CON, basal diet + saline; COND, DMG-Na-supplemented diet + saline; LPS, basal diet + LPS; LPSD, DMG-Na-supplemented diet + LPS. Values are means ± SD (n = 10 birds per treatment). *P*_D_, main effect of DMG-Na; *P*_L_, main effect of LPS; *P*_D×L_, interaction. Different lowercase letters indicate *P* < 0.05.

### Expression of selected hepatic genes

Hepatic *SIRT1, NF-κB P65, CLDN2*, and *CLDN3* gene expression were greater in LPS than in CON birds and lower in LPSD than in LPS birds (Fig. 8A). *SIRT1* gene expression was also lower in COND than in CON. In contrast, *SOD, GSH-Px, MnSOD*, and *γ-GCL* gene expression were lower in LPS than in CON and higher in LPSD than in LPS. Significant dietary DMG-Na × LPS interactions were detected for these gene expression (*P* < 0.05). The expression profiles of *OCLN* and *ZO1* are also shown in Fig. 8A. Among the mitochondrial-associated genes, *NDUFA2* exhibited a significant interaction (*P* = 0.048), with greater expression in COND than in CON and in LPSD than in LPS (Fig. 8B). No significant interaction was detected for the remaining mitochondrial-associated genes.

**Fig. 8:**
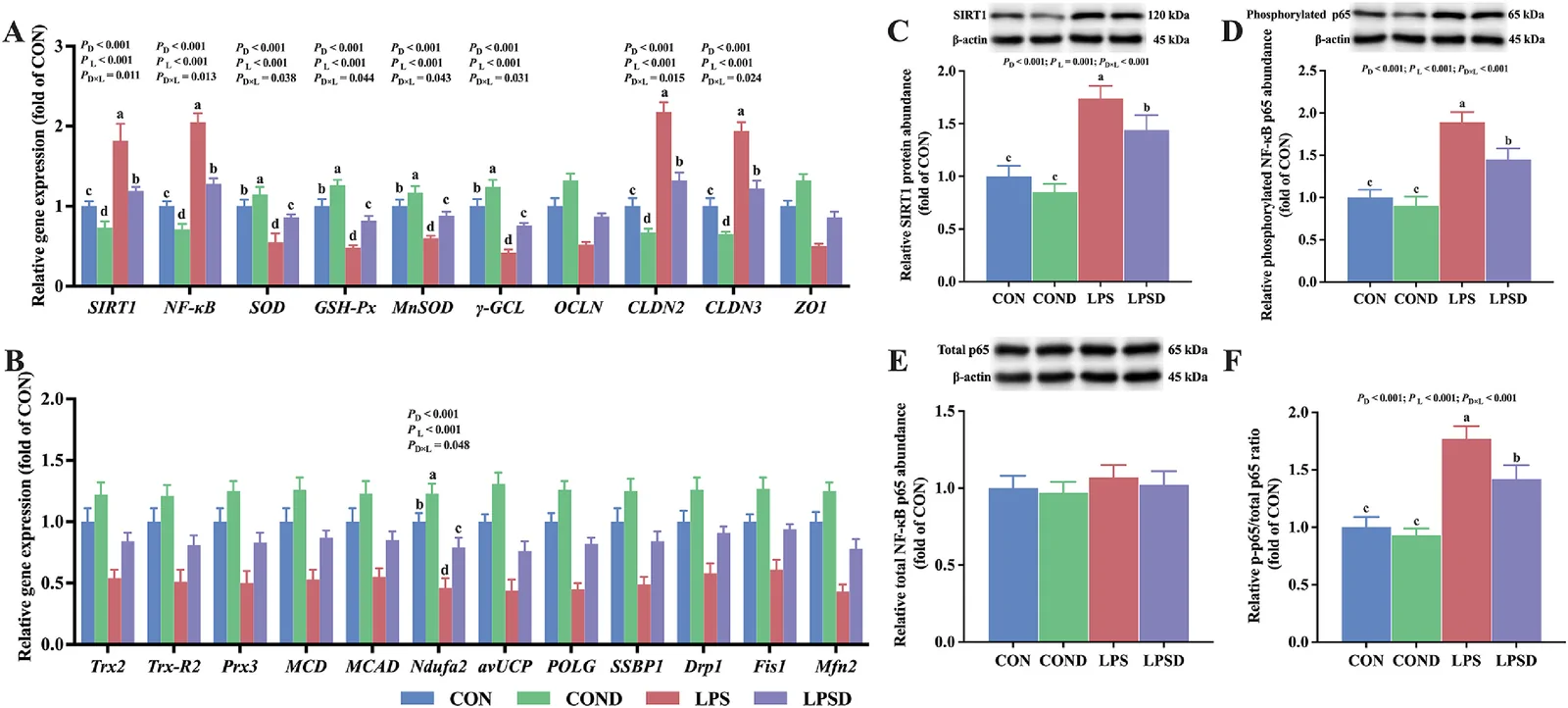
Hepatic gene expression, SIRT1 protein, and NF-κB p65 measurements in broilers supplemented with dimethylglycine sodium salt (DMG-Na) and challenged with lipopolysaccharide (LPS). (A) Selected inflammatory-, redox-, and junction-associated genes; (B) selected mitochondrial-associated genes; (C) SIRT1 protein abundance normalized to β-actin; (D) phosphorylated NF-κB p65 (Ser536) abundance normalized to β-actin; (E) total NF-κB p65 abundance normalized to β-actin; and (F) the phosphorylated-to-total p65 ratio calculated from matched measurements for each biological sample. Protein abundances and phosphorylation ratios are expressed relative to the corresponding CON group mean. Beta-actin (*β-actin*), sirtuin 1 (*SIRT1*), nuclear factor κB p65 (*NF-κB*), superoxide dismutase (*SOD*), glutathione peroxidase (*GSH-Px*), manganese superoxide dismutase (*MnSOD*), glutamate-cysteine ligase (*γ-GCL*), occludin (*OCLN*), claudin 2 (*CLDN2*), claudin 3 (*CLDN3*), zonula occludens-1 (*ZO1*), thioredoxin 2 (*Trx2*), thioredoxin reductase 2 (*Trx-R2*), peroxiredoxin 3 (*Prx3*), malonyl-CoA decarboxylase (*MCD*), medium-chain acyl-CoA dehydrogenase (*MCAD*), NADH:ubiquinone oxidoreductase subunit A2 (*NDUFA2*), avian uncoupling protein (*avUCP*), DNA polymerase gamma catalytic subunit (*POLG*), single-stranded DNA-binding protein 1 (*SSBP1*), dynamin-related protein 1 (*Drp1*), mitochondrial fission 1 (*Fis1*), and mitofusin 2 (*Mfn2*). CON, basal diet + saline; COND, DMG-Na-supplemented diet + saline; LPS, basal diet + LPS; LPSD, DMG-Na-supplemented diet + LPS. Means ± SD are shown (n = 10 birds per treatment). *P*_D_, dietary DMG-Na main effect; *P*_L_, LPS main effect; *P*_D×L_, interaction. Different lowercase letters indicate differences at *P* < 0.05.

### Hepatic SIRT1 abundance and NF-κB p65 phosphorylation

Hepatic SIRT1 protein abundance showed significant effects of dietary DMG-Na (*P* < 0.001), LPS challenge (*P* = 0.001), and their interaction (*P* < 0.001; Fig. 8C). Compared with CON, SIRT1 protein abundance was greater in LPS (*P* < 0.05) and numerically lower in COND (*P* > 0.05). The LPSD group had lower SIRT1 protein abundance than the LPS group (*P* < 0.05), although its abundance remained above that of CON (*P* < 0.05). The *SIRT1* gene expression showed the same direction of change across the treatment groups (Fig. 8A). Total NF-κB p65 protein abundance did not differ significantly among treatments (Fig. 8E). Phosphorylated NF-κB p65 (Ser536) abundance and the phosphorylated-to-total p65 ratio exhibited significant dietary DMG-Na × LPS interactions (both *P* < 0.001; Fig. 8D,F). Both indices were greater in LPS than in the saline-treated groups and lower in LPSD than in LPS (*P* < 0.05).

### Hepatic transcriptomic response

RNA sequencing of 40 liver samples generated 6.11∼9.63 Gb of clean data per library, with Q30 percentages of 94.18%∼95.11%, total mapping rates of 93.56%∼95.25%, and uniquely mapped rates of 88.2%∼90.9% (Table S2). Principal-component analysis of the transcriptomic profiles is shown in Fig. 9A. The factorial DESeq2 analysis identified 138 genes with significant DMG-Na × LPS interactions (Benjamini-Hochberg-adjusted *P* < 0.05), including 54 positive and 84 negative interaction coefficients (Fig. 9B).

**Fig. 9:**
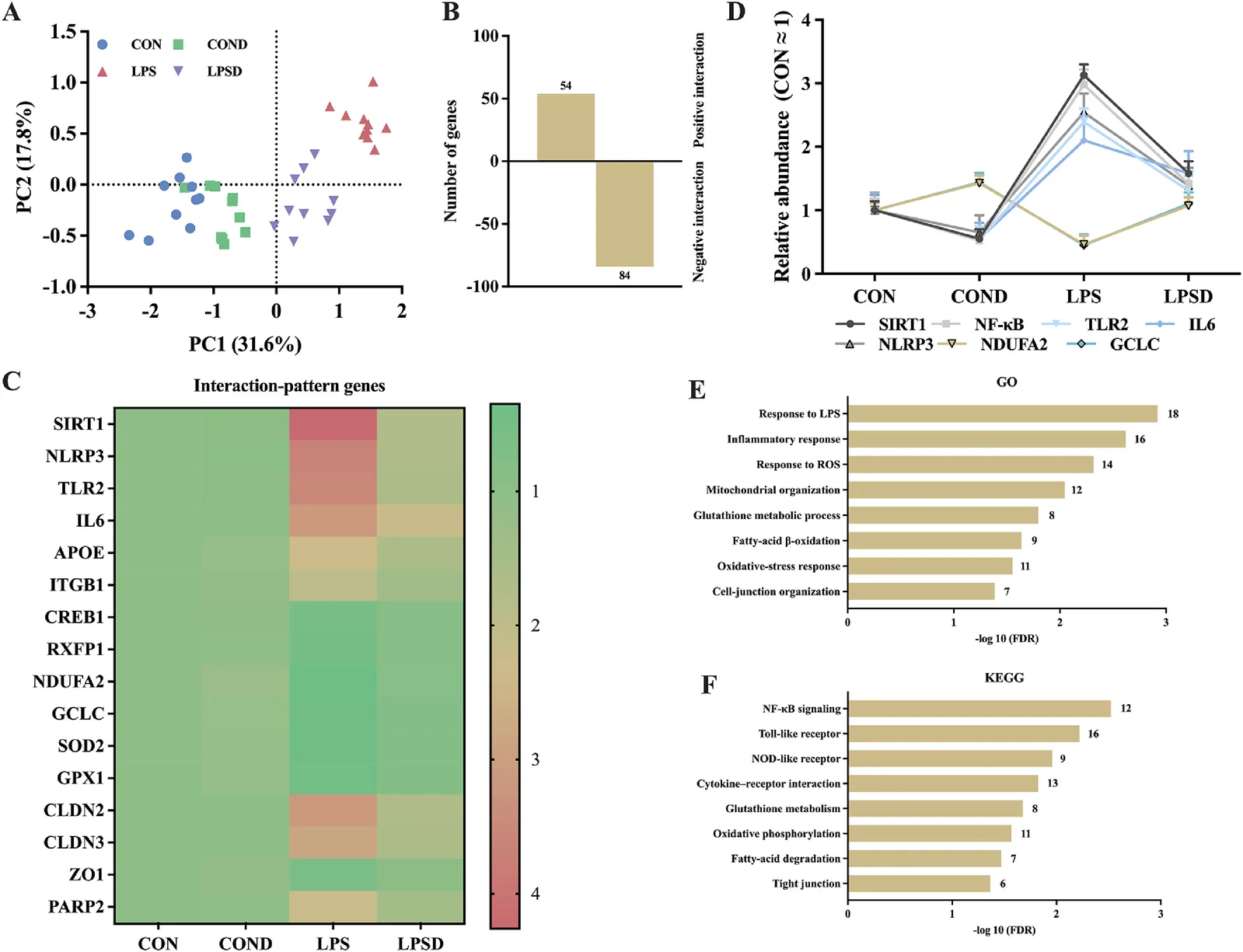
Factorial hepatic RNA-sequencing analysis in broilers given dietary dimethylglycine sodium salt (DMG-Na) and lipopolysaccharide (LPS). (A) Principal-component analysis of normalized transcriptomes; (B) numbers of significant positive and negative DMG-Na × LPS interaction coefficients; (C) heatmap of selected interaction-associated genes; (D) expression profiles of selected genes; (E) Gene Ontology biological-process enrichment; and (F) KEGG pathway enrichment. The interaction was defined as (LPSD - LPS) - (COND - CON) and tested in DESeq2, with Benjamini-Hochberg-adjusted *P* < 0.05. CON, basal diet + saline; COND, DMG-Na-supplemented diet + saline; LPS, basal diet + LPS; LPSD, DMG-Na-supplemented diet + LPS. RNA-seq included 10 independent birds per treatment (n = 40).

Among the interaction-associated genes, negative interaction log₂ fold changes were observed for *NLRP3, NF-κB p65, TLR2, IL6*, and *SIRT1*. Positive coefficients were identified for *NDUFA2* and *GCLC* (Fig. 9C, D and Table S3). Gene Ontology enrichment included inflammatory responses, responses to LPS and reactive oxygen species, mitochondrial organization, and glutathione- and lipid-related processes (Fig. 9E). KEGG enrichment included Toll-like receptor, NF-κB, NOD-like receptor, glutathione metabolism, and oxidative phosphorylation pathways (Fig. 9F). In the secondary pairwise contrasts, 324 differentially expressed genes (DEGs) were identified for COND versus CON (212 higher and 112 lower), 826 for LPS versus CON (484 higher and 342 lower), and 364 for LPSD versus LPS (215 higher and 149 lower), using adjusted *P* < 0.05 and |log₂ fold change| ≥ 2 (Table S4A). Additional interaction and simple-effect estimates and pairwise enrichment results are provided in Tables S3 and S4B.

### Hepatic metabolomic response

Untargeted hepatic metabolomics detected 8,946 features in positive electrospray ionization (ESI+) mode and 6,318 in negative (ESI-) mode. After missingness and pooled quality-control filtering, 7,604 ESI+ features and 5,491 ESI- features remained for statistical analysis (Table S5). The median pooled-QC relative standard deviations were 11.6% and 10.8% in ESI+ and ESI- modes, respectively. Sample ordination is shown in Fig. 10A, while pooled quality-control (QC) signal profiles and annotation confidence are reported in Tables S8 and S6A-B.

**Fig. 10:**
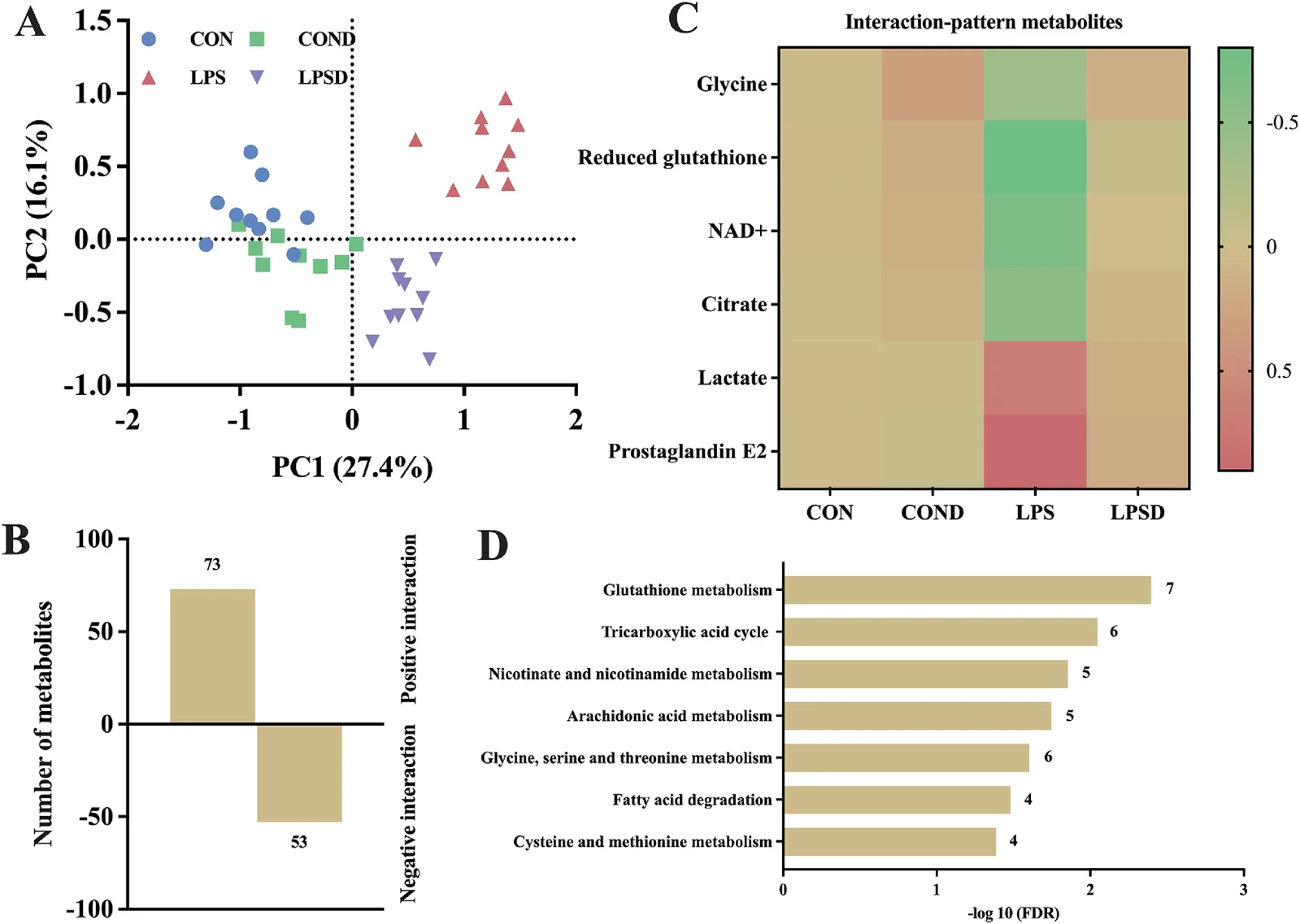
Hepatic untargeted metabolomics in broilers receiving dietary dimethylglycine sodium salt (DMG-Na) and lipopolysaccharide (LPS). (A) Principal-component analysis of normalized features; (B) numbers of features with positive or negative DMG-Na × LPS interaction coefficients; (C) abundance patterns of selected putatively annotated metabolites; and (D) enrichment of metabolic pathways associated with interaction-responsive features. The interaction was defined as (LPSD - LPS) - (COND - CON), with Benjamini-Hochberg-adjusted *P* < 0.05 in feature-wise limma models. CON, basal diet + saline; COND, DMG-Na-supplemented diet + saline; LPS, basal diet + LPS; LPSD, DMG-Na-supplemented diet + LPS. Forty independent liver extracts were assessed (10 per treatment).

The factorial metabolomic analysis identified 126 features with significant DMG-Na × LPS interactions (adjusted *P* < 0.05), comprising 73 positive and 53 negative interaction coefficients (Fig. 10B). Features putatively annotated as reduced glutathione, NAD^+^, citrate, and glycine had positive interactions. Lactate and prostaglandin E2 had negative interactions (Fig. 10C and Table S9A). Pathway enrichment of interaction-associated metabolite features included glutathione metabolism, the tricarboxylic acid cycle, nicotinate and nicotinamide metabolism, glycine metabolism, cysteine metabolism, fatty-acid degradation, and arachidonic-acid metabolism (Fig. 10D). Secondary comparisons identified 96 differential features in COND versus CON, 214 in LPS versus CON, and 183 in LPSD versus LPS (adjusted *P* < 0.05, fold change > 2.0 or < 0.5, Table S7A). The factorial coefficients and secondary simple-effect estimates are reported in Tables S9A and S9B. Selected metabolite annotations were supported by spectral-library evidence at Metabolomics Standards Initiative Level 2 (Table S8).

### Breast-muscle quality

Breast-muscle drip loss exhibited significant effects of dietary DMG-Na (*P*_D_ = 0.006), LPS (*P*_L_ < 0.001), and their interaction (*P*_D×L_ = 0.041; Fig. 11C). Drip loss was lower in COND than in CON and was greatest in LPS. The LPSD group exhibited an intermediate value that did not differ significantly from either LPS or CON in Tukey-adjusted comparisons. Neither dietary DMG-Na supplementation nor LPS challenge, nor their interaction, significantly affected muscle pH at 24 h postmortem (*P* > 0.05; Fig. 11B). Similarly, muscle pH at 1 h postmortem, cooking loss, Warner–Bratzler shear force, and CIE L*, a*, and b* values did not differ significantly among treatment groups (Fig. 11A, D-H).

**Fig. 11:**
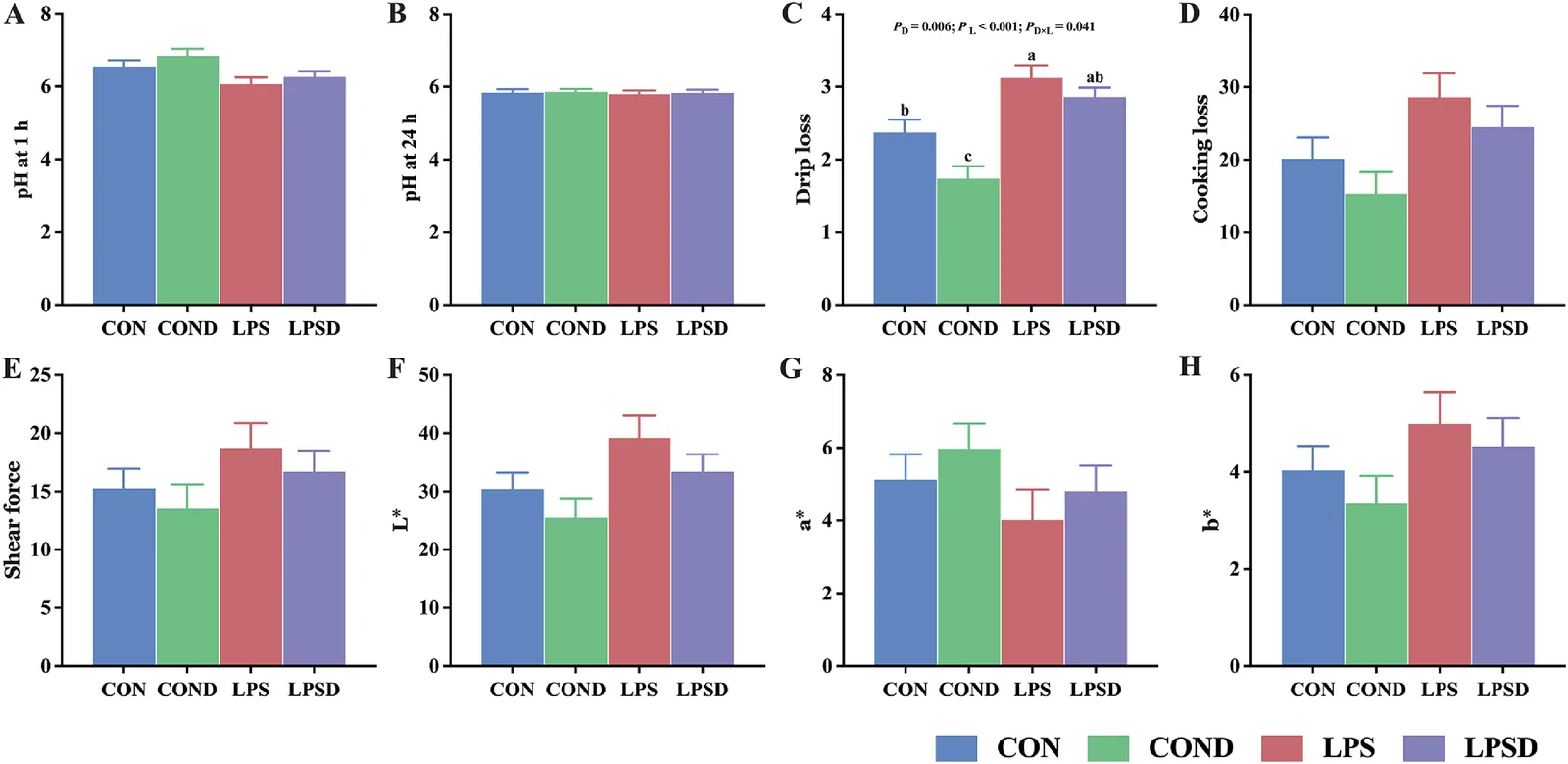
Breast-muscle quality of broilers given dietary dimethylglycine sodium salt (DMG-Na) and challenged with lipopolysaccharide (LPS). (A) pH at 1 h postmortem, (B) pH at 24 h postmortem, (C) drip loss, (D) cooking loss, (E) Warner-Bratzler shear force, (F) CIE lightness (L*), (G) redness (a*), and (H) yellowness (b*). CON, basal diet plus saline, COND, DMG-Na-supplemented diet plus saline, LPS, basal diet plus LPS, LPSD, DMG-Na-supplemented diet plus LPS. Values represent means ± SD from 10 independent birds per treatment, with one bird sampled from each replicate cage. *P*_D_, main effect of dietary DMG-Na, *P*_L_, main effect of LPS, *P*_D×L_, interaction effect. Different lowercase letters indicate significant between-treatment differences (*P* < 0.05).

### Associations between hepatic variables and breast-muscle drip loss

Among the eight selected hepatic and serum variables examined in separate treatment-adjusted linear models, SIRT1 gene expression was positively associated with continuous breast-muscle drip loss (coefficient per one-SD increase in the predictor, β = 0.41 percentage points; BH-FDR = 0.043; Fig. 12D and Table S11A). The adjusted coefficients for the NF-κB gene expression (β = 0.27), fraction-associated protein carbonyls (β = 0.25), and mtDNA abundance (β = −0.23) did not reach the FDR threshold. The remaining variables, NLRP3, NDUFA2, GSH-Px activity, and serum ALT activity, were likewise nonsignificant after adjustment (FDR > 0.05). The pooled Spearman correlation between hepatic SIRT1 gene expression and drip loss was ρ = 0.72 (*P* < 0.001; Table S11B). Within-treatment coefficients were ρ = 0.39 in CON, 0.31 in COND, 0.47 in LPS, and 0.36 in LPSD. None reached *P* < 0.05 (n = 10 per group; Tables S11A-B). Pooled correlations were also generally greater in magnitude than within-treatment correlations for the other variables examined.

**Fig. 12:**
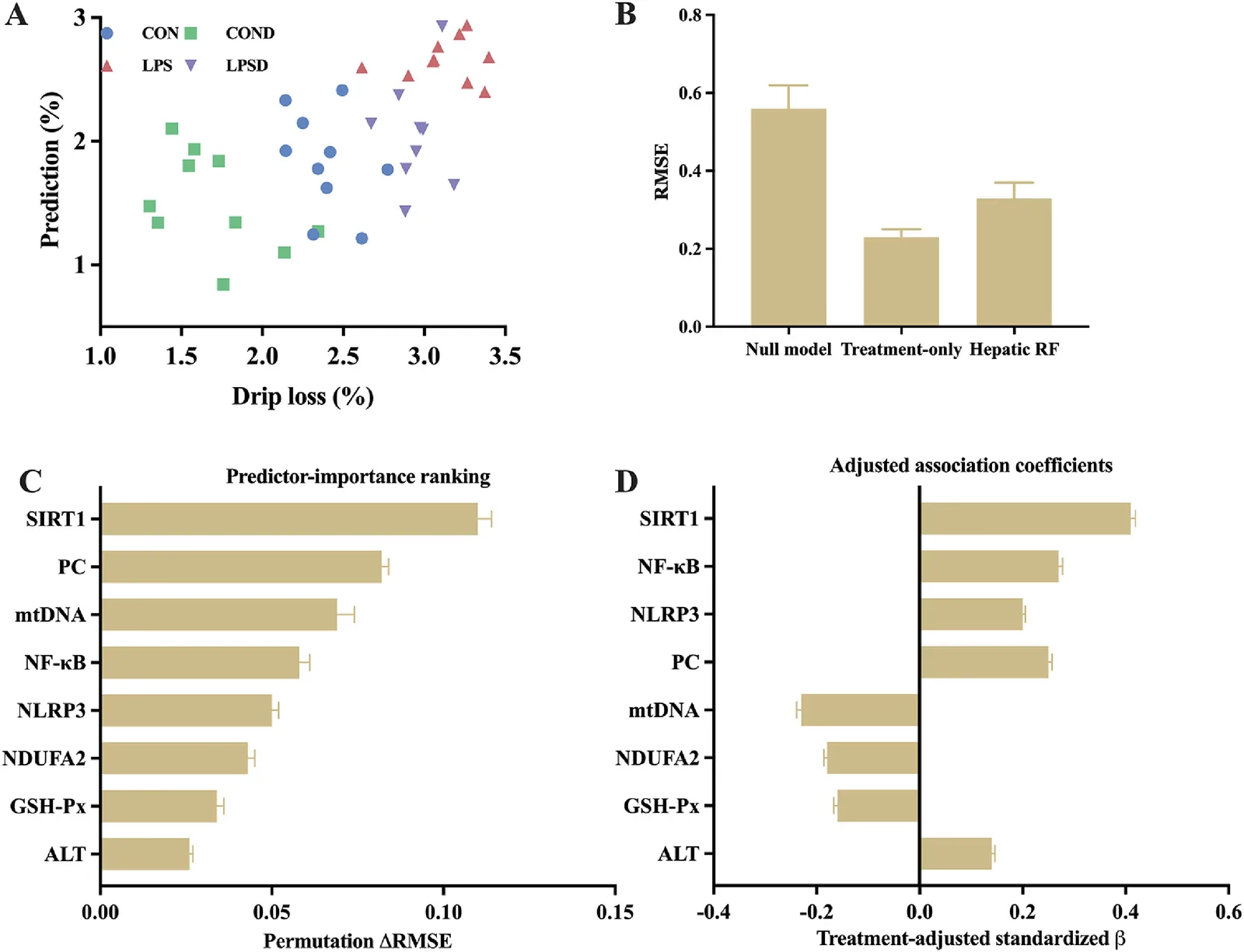
Treatment-adjusted liver-muscle associations and nested cross-validated regression of continuous breast-muscle drip loss. (A) Observed versus out-of-fold predicted drip loss in the hepatic-variable random forest, the diagonal is the identity line. (B) Root mean square error (RMSE, percentage points) for the null, treatment-only, and hepatic-variable models, with error bars as displayed. (C) Permutation importance of hepatic predictors summarized across outer resampling iterations. (D) Regression coefficients adjusted for DMG-Na, LPS, and their interaction (percentage points of drip loss per predictor SD); 95% confidence intervals are reported in Table S11A. Each outer fold was stratified to include two birds from each treatment group, and the cross-validation procedure was repeated 10 times. Predictor selection and hyperparameter tuning were performed exclusively within the training folds. The matched dataset included 40 birds (10 per treatment).

### Exploratory prediction of continuous drip loss

Repeated nested cross-validation of the hepatic-variable random-forest model yielded an out-of-fold RMSE of 0.33 percentage points, an MAE of 0.25 percentage points, and an R² of 0.64. The relationship between observed and predicted drip loss is presented in Fig. 12A. The treatment-only model achieved the lowest RMSE (0.23 percentage points), followed by the hepatic-variable random forest (0.33 percentage points) and the null model (0.56 percentage points, Fig. 12B). The treatment-only model achieved an R² of 0.84. SIRT1 gene expression, mitochondria-enriched fraction protein carbonyls, and mtDNA abundance ranked highest in permutation importance (Fig. 12C). Treatment-adjusted regression coefficients are displayed in Fig. 12D, with their 95% confidence intervals reported in Table S11A.

## Discussion

Dietary DMG-Na supplementation attenuated the reduction in growth performance induced by repeated LPS challenge, with higher postchallenge ADG and lower FCR in LPSD than in LPS birds. Because ADFI did not differ among treatments, the higher ADG and lower FCR indicate more efficient utilization of consumed feed in supplemented challenged birds. Immune activation imposes competing demands on nutrient utilization through inflammatory and acute-phase responses, contributing to reduced growth efficiency in challenged broilers (Shi et al., 2022, and Zhi et al., 2024). Dimethylglycine is an intermediate in the conversion of betaine to sarcosine and is linked to one-carbon and glycine metabolism, providing a biochemical basis for investigating its effects on metabolic adaptation during immune challenge. Previous studies have documented performance and metabolic responses to dietary dimethylglycine in broilers (Kalmar et al., 2011), including improvements during heat stress (Bai et al., 2026). The present factorial trial extends these findings to repeated endotoxin exposure and identifies a challenge-dependent effect of 0.4 g DMG-Na/kg basal diet on growth efficiency.

Dietary DMG-Na also modified circulating lipids in LPS-challenged broilers. LPS increased circulating TC and TG, whereas both indices were lower in supplemented challenged birds. As the liver plays a central role in avian lipid synthesis and transport, these changes are relevant to the hepatic response to inflammatory stress. Previous work has linked DMG-Na supplementation to lipid-related metabolic regulation in growth restricted pigs (Bai et al., 2022, 2024), and the present results demonstrate a corresponding response in broilers exposed to LPS. Serum glucose, LDL-C, and HDL-C remained unchanged, indicating that the circulating metabolic response was most apparent in TC and TG.

Quantitative histology and transmission electron microscopy provided direct tissue-level evidence that DMG-Na attenuated LPS-associated hepatic alterations. Supplementation reduced the severity of hepatocellular lesions and lowered the proportions of mitochondrial profiles exhibiting swelling and disrupted cristae. The parallel decreases in serum ALT, AST, ALP, and LDH activities are consistent with an improved systemic biochemical profile, although these enzymes are not specific to hepatic tissue in birds (Franson et al., 1985; Siska et al., 2017). The tissue-level findings are consistent with attenuation of LPS-associated hepatic structural damage. Comparable improvements in hepatic morphology and mitochondrial ultrastructure have been reported following nutritional interventions in chickens exposed to inflammatory or oxidative challenges (Yuan et al., 2025; Zhan et al., 2025). Although lesions remained detectable after supplementation, their lower severity and the reduced prevalence of mitochondrial abnormalities demonstrate attenuation of the structural response to LPS.

Protein carbonyl concentrations in mitochondria-enriched liver fractions were lower in LPSD than in LPS birds, coinciding with reduced mitochondrial swelling and cristae abnormalities. Protein carbonylation reflects oxidative modification of cellular proteins and provides information distinct from lipid peroxidation or antioxidant enzyme activity. Serum SOD exhibited treatment-dependent changes, whereas most whole-liver redox indices and several activities in the enriched fractions remained unchanged. The reduction in protein carbonyl content was more pronounced than the changes in the measured antioxidant enzyme activities. Relative hepatic mtDNA abundance was higher in LPSD than in LPS birds, accompanying the more favorable mitochondrial morphology and the lower fraction-associated protein carbonyl content. However, hepatic ATP concentration and the measured activities of respiratory-chain complexes I-V did not differ among treatments. In heat-stressed broilers, DMG-Na supplementation has been associated with changes in mitochondrial respiratory-chain activity and ATP production (Bai et al., 2026). The difference between these findings and the present LPS-challenge results illustrates the dependence of mitochondrial responses on the physiological challenge and the endpoints examined. These results identify mitochondrial ultrastructure and relative mtDNA abundance as principal hepatic endpoints responsive to DMG-Na during LPS challenge.

The hepatic SIRT1 and NF-κB measurements revealed coordinated responses to repeated LPS exposure and dietary DMG-Na supplementation. Both SIRT1 gene and protein abundances increased following LPS challenge, whereas supplementation reduced their abundance in challenged birds. SIRT1 protein was also numerically lower in COND than in CON, consistent with the overall direction of the dietary response. LPS simultaneously increased phosphorylated p65 abundance and the phosphorylated-to-total p65 ratio, and DMG-Na reduced both indices in challenged birds without a significant change in total p65 abundance. NF-κB p65 phosphorylation and its abundance are associated with inflammatory transcriptional responses. The concurrent elevation of SIRT1 gene expression and p65 activation-related indices under LPS challenge indicates that changes in SIRT1 protein abundance cannot be interpreted as an inverse measure of NF-κB signaling. SIRT1 has established roles in NAD^+^-dependent regulation of inflammatory transcription (Yeung et al., 2004; de Gregorio et al., 2020). The present observations identify coordinated dietary changes in SIRT1 expression and NF-κB-associated protein responses during LPS challenge, alongside the improvements in hepatic morphology and redox-related indices.

The factorial transcriptomic analysis places these targeted measurements within a broader hepatic regulatory response. Among 138 genes showing significant dietary DMG-Na × LPS interactions, NLRP3, NF-κB, TLR2, and IL6 displayed negative interaction coefficients, whereas GCLC and NDUFA2 displayed positive coefficients. These genes connect innate immune sensing and inflammatory signaling with glutathione biosynthesis, oxidative defense, and mitochondrial biology. The enrichment of Toll-like receptor, NF-κB, NOD-like receptor, glutathione-metabolism, and mitochondrial-associated pathways was consistent with the tissue, redox, and p65 protein results. These interaction-associated changes differed between LPS-challenged and saline-treated birds. The negative interaction coefficient for SIRT1 was consistent with attenuation of the LPS-associated increase in SIRT1 expression after dietary supplementation, as observed in the targeted gene and protein measurements. Collectively, the interaction-associated genes and targeted protein indices characterize the hepatic inflammatory and oxidative responses accompanying DMG-Na supplementation.

Untargeted metabolomics provided complementary evidence of hepatic metabolic remodeling. Positive DMG-Na × LPS interaction coefficients were observed for features putatively annotated as reduced glutathione, NAD^+^, glycine, and citrate, whereas lactate and prostaglandin E2 displayed negative coefficients. These compounds are associated with glutathione-dependent redox balance, one-carbon metabolism, central carbon metabolism, and inflammatory lipid signaling. These metabolic features were concordant with transcriptomic enrichment of glutathione-related and inflammatory pathways and with the lower p65 activation-related indices in supplemented challenged birds. NAD^+^ is a biochemical substrate for SIRT1-dependent deacetylation, but the metabolomic feature abundance and SIRT1 protein measurements represent distinct endpoints. N,N-Dimethylglycine itself showed a pronounced supplementation-associated simple effect but little factorial interaction, separating the dietary exposure response from the broader challenge-dependent metabolic profile. The metabolomic annotations were supported by spectral-library matching at MSI Level 2, and the observed feature changes characterize metabolite abundance rather than metabolic flux. These metabolomic profiles align with the glutathione-related transcriptomic response and the targeted measurements of hepatic redox indices.

Dietary DMG-Na had more pronounced effects on hepatic endpoints than on breast-muscle quality. Drip loss exhibited a significant dietary DMG-Na × LPS interaction, but the direct comparison between LPSD and LPS was not significant, and neither postmortem pH nor the other measured meat-quality traits differed among treatments. Postmortem water retention is influenced by muscle glycolysis and protein structure (Przybylski et al., 2022; Zhang et al., 2022), but these processes were not directly linked to drip loss in the present study. The significant interaction alone does not establish that DMG-Na reduced drip loss in LPS-challenged birds. Although hepatic SIRT1 gene expression was associated with drip loss in the pooled and treatment-adjusted analyses, correlations within individual treatments were weaker. The treatment-only model had greater predictive accuracy than the hepatic-variable model, indicating a substantial contribution of treatment allocation to the observed associations. Hepatic and breast-muscle outcomes therefore showed different response patterns.

Overall, supplementation with 0.4 g DMG-Na/kg basal diet improved postchallenge growth efficiency and attenuated hepatic histological and mitochondrial alterations in LPS-challenged broilers. The accompanying decreases in fraction-associated protein carbonyl content, hepatic SIRT1 protein abundance, and phosphorylated NF-κB p65 abundance, together with the factorial omics changes, characterize the inflammatory and redox-related responses to supplementation in LPS-challenged birds. By integrating performance, quantitative hepatic morphology, protein abundance and phosphorylation measurements, and factorial multi-omics analysis, the study extends evidence for DMG-Na to repeated immune challenge in broiler chickens. These findings support further evaluation of dietary DMG-Na as a candidate nutritional intervention for attenuating hepatic structural and inflammatory-redox alterations during repeated LPS challenge.

## Author contributions

Luyi Jiang contributed to the study design, conducted the animal trial, collected biological samples, performed laboratory analyses, interpreted the results, and drafted the manuscript. Yaqi Rao participated in the animal trial, biological sample collection, laboratory analyses, and data analysis, and critically reviewed and revised the manuscript for important intellectual content. Kaiwen Bai conceived and designed the study, supervised the research, contributed to data analysis and interpretation, and critically revised the manuscript for important intellectual content. All authors have reviewed and approved the final version of the manuscript for publication and agree to be accountable for all aspects of the work, ensuring that any questions regarding the accuracy or integrity of the research are appropriately investigated and resolved.

## Disclosures

The authors declare that there is no conflict of interest.

## Data Availability

The datasets generated and analyzed in this study, including the RNA-sequencing and metabolomic data are available from the corresponding author upon reasonable request.

## Footnotes

Supplementary material associated with this article can be found, in the online version, at doi:10.1016/j.psj.2026.107994.
