# Supplementary material for: Dietary dimethylglycine sodium salt attenuates lipopolysaccharide-associated hepatic alterations and modulates redox responses in broiler chickens
Source: Poult Sci. 2026 Oct 20;105(12):107994. doi: 10.1016/j.psj.2026.107994 (PMC13718808; doi:10.1016/j.psj.2026.107994)
Supplement: Supplementary file 1 [file mmc1.docx]

**Supplementary tables**

**Table S1. Experimental diet composition and calculated nutrients (as-fed basis)**

| Item^1^ | Basal diet (d 1-21) |
| --- | --- |
| Ingredients (%) |  |
| Corn | 54.79 |
| Soybean meal | 32.70 |
| Corn gluten meal | 4.50 |
| Soybean oil | 3.20 |
| Dicalcium phosphate | 1.80 |
| Limestone | 1.30 |
| L-Lysine | 0.28 |
| DL-Methionine | 0.13 |
| Salt | 0.30 |
| Premix^2^ | 1.00 |
| Total | 100.00 |
| Calculated composition |  |
| Metabolisable energy (MJ/kg) | 12.55 |
| Crude protein | 22.07 |
| Calcium | 1.00 |
| Available phosphorus | 0.43 |
| Lysine | 1.20 |
| Methionine | 0.50 |
| Methionine + cysteine | 0.87 |

^1^ Ingredient inclusion rates are expressed as parts by weight per 100 parts of the basal diet, rather than as percentages of the final supplemented mixture. The additional DMG-Na is not included in the 100.00% basal ingredient total. Metabolizable energy is expressed in MJ/kg, and other calculated nutrient concentrations are expressed as percentages unless otherwise indicated. The basal diet was formulated for broilers from d 1 to 21. Dimethylglycine sodium salt (DMG-Na, 99.9% purity) was added at 0.4 g per kg basal diet without replacing any ingredient.

^2^ Premix supplied the following quantities per kg of diet: vitamin A (transretinyl acetate, 10,000 IU), vitamin D3 (cholecalciferol, 3,000 IU), vitamin E (all-rac-α-tocopherol acetate, 30 IU), menadione (1.3 mg), thiamin (2.2 mg), riboflavin (8 mg), nicotinamide (40 mg), choline chloride (600 mg), calcium pantothenate (10 mg), pyridoxine hydrochloride (4 mg), biotin (0.04 mg), folic acid (1 mg), vitamin B12 (cobalamin, 0.013 mg), Fe (ferrous sulfate, 80 mg), Cu (copper sulfate, 8 mg), Mn (manganese sulfate, 110 mg), iodine (calcium iodate, 1.1 mg), and Se (sodium selenite, 0.3 mg).

**Table S2. Quality-control and mapping statistics for liver RNA sequencing**

| Sample | Treatment | RIN | Raw reads | Clean reads | Clean bases (Gb) | Q20 (%) | Q30 (%) | GC (%) | Total mapped (%) | Uniquely mapped (%) | Assigned fragments (%) |
| --- | --- | --- | --- | --- | --- | --- | --- | --- | --- | --- | --- |
| CON01 | CON | 8.3 | 55,486,000 | 54,909,000 | 8.16 | 98.28 | 95.03 | 50.02 | 94.74 | 90.20 | 84.49 |
| CON02 | CON | 8.3 | 49,532,000 | 48,936,000 | 7.26 | 97.64 | 94.64 | 49.29 | 94.31 | 90.05 | 83.65 |
| CON03 | CON | 9.0 | 55,312,000 | 54,794,000 | 8.15 | 97.66 | 94.42 | 49.19 | 93.80 | 90.17 | 85.14 |
| CON04 | CON | 7.9 | 40,953,000 | 40,700,000 | 6.11 | 97.94 | 94.99 | 50.15 | 94.39 | 88.74 | 82.28 |
| CON05 | CON | 7.6 | 51,680,000 | 51,311,000 | 7.62 | 98.02 | 94.90 | 50.33 | 94.97 | 88.58 | 84.30 |
| CON06 | CON | 9.2 | 53,619,000 | 53,092,000 | 7.96 | 97.55 | 94.53 | 50.27 | 94.34 | 89.56 | 83.17 |
| CON07 | CON | 9.1 | 59,833,000 | 59,366,000 | 8.81 | 97.07 | 94.27 | 49.35 | 94.55 | 90.94 | 85.79 |
| CON08 | CON | 7.9 | 63,159,000 | 62,665,000 | 9.38 | 97.34 | 94.36 | 48.71 | 94.70 | 89.40 | 84.20 |
| CON09 | CON | 7.5 | 52,250,000 | 51,787,000 | 7.76 | 97.57 | 94.47 | 50.26 | 94.20 | 89.80 | 83.67 |
| CON10 | CON | 7.4 | 60,852,000 | 59,996,000 | 8.91 | 97.90 | 94.99 | 48.98 | 94.73 | 89.22 | 83.87 |
| COND01 | COND | 8.1 | 62,959,000 | 62,242,000 | 9.31 | 97.32 | 94.41 | 48.82 | 94.35 | 89.44 | 83.75 |
| COND02 | COND | 8.6 | 63,952,000 | 63,501,000 | 9.46 | 97.27 | 94.18 | 49.38 | 95.20 | 88.59 | 84.57 |
| COND03 | COND | 7.5 | 61,868,000 | 60,998,000 | 9.15 | 97.28 | 94.25 | 50.52 | 93.83 | 90.92 | 85.98 |
| COND04 | COND | 9.1 | 52,718,000 | 52,011,000 | 7.77 | 97.78 | 94.64 | 50.64 | 93.88 | 88.29 | 83.84 |
| COND05 | COND | 8.1 | 42,826,000 | 42,291,000 | 6.30 | 97.42 | 94.43 | 49.41 | 93.56 | 90.42 | 85.87 |
| COND06 | COND | 8.9 | 59,990,000 | 59,558,000 | 8.89 | 97.04 | 94.24 | 49.87 | 94.15 | 90.50 | 84.74 |
| COND07 | COND | 8.4 | 51,480,000 | 50,773,000 | 7.56 | 98.05 | 94.73 | 49.29 | 93.99 | 89.44 | 84.71 |
| COND08 | COND | 7.5 | 59,169,000 | 58,313,000 | 8.67 | 97.90 | 94.78 | 49.87 | 94.51 | 89.14 | 82.78 |
| COND09 | COND | 8.4 | 51,063,000 | 50,329,000 | 7.51 | 97.79 | 94.85 | 50.25 | 94.88 | 90.30 | 85.32 |
| COND10 | COND | 8.8 | 42,483,000 | 42,077,000 | 6.29 | 97.99 | 94.97 | 50.09 | 93.85 | 88.15 | 83.63 |
| LPS01 | LPS | 9.2 | 57,902,000 | 57,239,000 | 8.54 | 97.95 | 94.88 | 49.08 | 93.61 | 90.86 | 85.64 |
| LPS02 | LPS | 9.0 | 44,334,000 | 43,743,000 | 6.52 | 97.91 | 94.57 | 49.23 | 94.24 | 88.36 | 82.51 |
| LPS03 | LPS | 8.1 | 53,117,000 | 52,398,000 | 7.84 | 97.80 | 94.73 | 49.89 | 94.87 | 88.23 | 84.14 |
| LPS04 | LPS | 8.2 | 58,522,000 | 58,024,000 | 8.64 | 97.52 | 94.57 | 50.74 | 94.05 | 88.20 | 83.94 |
| LPS05 | LPS | 7.7 | 64,320,000 | 63,828,000 | 9.49 | 98.10 | 95.03 | 50.49 | 94.80 | 88.30 | 83.49 |
| LPS06 | LPS | 8.6 | 59,584,000 | 58,876,000 | 8.82 | 97.97 | 94.82 | 50.48 | 95.17 | 88.53 | 84.23 |
| LPS07 | LPS | 8.8 | 42,890,000 | 42,305,000 | 6.28 | 97.83 | 94.83 | 48.99 | 94.34 | 89.53 | 83.80 |
| LPS08 | LPS | 7.7 | 65,452,000 | 64,700,000 | 9.63 | 97.82 | 95.01 | 48.88 | 94.33 | 89.65 | 83.54 |
| LPS09 | LPS | 7.7 | 42,195,000 | 41,677,000 | 6.24 | 97.47 | 94.52 | 49.00 | 94.39 | 89.16 | 84.56 |
| LPS10 | LPS | 9.0 | 54,905,000 | 54,226,000 | 8.04 | 97.59 | 94.66 | 49.31 | 94.00 | 90.37 | 84.30 |
| LPSD01 | LPSD | 8.8 | 49,758,000 | 49,242,000 | 7.37 | 97.46 | 94.32 | 49.25 | 93.95 | 89.29 | 83.18 |
| LPSD02 | LPSD | 8.4 | 44,560,000 | 44,237,000 | 6.64 | 98.43 | 95.11 | 49.87 | 95.13 | 88.38 | 83.09 |
| LPSD03 | LPSD | 9.0 | 45,105,000 | 44,789,000 | 6.66 | 97.63 | 94.69 | 50.18 | 94.18 | 89.42 | 84.97 |
| LPSD04 | LPSD | 7.6 | 49,225,000 | 48,700,000 | 7.24 | 98.13 | 94.91 | 49.01 | 95.18 | 89.09 | 83.48 |
| LPSD05 | LPSD | 9.2 | 42,526,000 | 42,062,000 | 6.30 | 97.61 | 94.44 | 50.66 | 95.25 | 90.35 | 86.14 |
| LPSD06 | LPSD | 8.5 | 58,763,000 | 58,292,000 | 8.65 | 97.80 | 94.70 | 49.00 | 94.54 | 89.35 | 84.91 |
| LPSD07 | LPSD | 8.8 | 44,376,000 | 44,112,000 | 6.57 | 97.14 | 94.31 | 50.66 | 93.74 | 88.64 | 83.14 |
| LPSD08 | LPSD | 9.4 | 59,691,000 | 59,056,000 | 8.79 | 97.57 | 94.40 | 50.44 | 95.19 | 90.22 | 84.69 |
| LPSD09 | LPSD | 8.0 | 48,090,000 | 47,478,000 | 7.07 | 97.81 | 94.48 | 50.11 | 94.20 | 90.44 | 84.32 |
| LPSD10 | LPSD | 8.9 | 61,799,000 | 61,261,000 | 9.19 | 97.71 | 94.56 | 50.18 | 94.28 | 90.86 | 84.38 |

Forty independent liver RNA samples were analyzed (10 birds per treatment, one per replicate cage). RIN denotes RNA integrity number. Q20 and Q30 represent the percentages of bases with Phred scores ≥20 and ≥30, respectively. GC denotes guanine–cytosine content. Raw and clean reads refer to reads before and after quality filtering. Clean bases report filtered sequence yield (Gb). Total and unique mapping refer to alignment with bGalGal1.mat.broiler.GRCg7b (GCF_016699485.2). Assigned fragments are paired-end fragments counted using featureCounts. CON, basal diet + saline, COND, DMG-Na-supplemented diet + saline, LPS, basal diet + LPS, LPSD, DMG-Na-supplemented diet + LPS.

**Table S3. Selected RNA-seq factorial interaction and secondary simple-effect contrasts**

| Gene | COND vs. CON log₂FC | FDR | LPS vs. CON log₂FC | FDR | LPSD vs. LPS log₂FC | FDR | LPSD vs. COND log₂FC | FDR | Interaction log₂FC | Interaction FDR |
| --- | --- | --- | --- | --- | --- | --- | --- | --- | --- | --- |
| SIRT1 | -0.10 | 0.742 | 1.70 | 0.001 | -0.98 | 0.006 | 0.82 | 0.014 | -0.88 | 0.018 |
| NF-κB | -0.13 | 0.360 | 1.30 | 0.005 | -0.98 | 0.005 | 0.45 | 0.077 | -0.85 | 0.009 |
| NLRP3 | -0.15 | 0.641 | 2.20 | <0.001 | -1.47 | 0.004 | 0.88 | 0.018 | -1.32 | 0.004 |
| TLR2 | -0.05 | 0.812 | 1.80 | 0.002 | -1.15 | 0.008 | 0.70 | 0.031 | -1.10 | 0.011 |
| IL6 | 0.02 | 0.936 | 2.60 | <0.001 | -1.46 | 0.002 | 1.12 | 0.006 | -1.48 | 0.002 |
| APOE | 0.18 | 0.383 | 1.20 | 0.009 | -0.54 | 0.110 | 0.48 | 0.130 | -0.72 | 0.041 |
| ITGB1 | 0.12 | 0.487 | 0.95 | 0.018 | -0.54 | 0.090 | 0.29 | 0.290 | -0.66 | 0.039 |
| CREB1 | 0.05 | 0.798 | -0.90 | 0.021 | 0.73 | 0.037 | -0.22 | 0.370 | 0.68 | 0.042 |
| RXFP1 | 0.09 | 0.615 | -1.05 | 0.012 | 0.83 | 0.025 | -0.31 | 0.250 | 0.74 | 0.037 |
| NDUFA2 | 0.35 | 0.130 | -1.55 | 0.003 | 1.31 | 0.004 | -0.59 | 0.080 | 0.96 | 0.015 |
| GCLC | 0.22 | 0.290 | -1.40 | 0.004 | 1.05 | 0.009 | -0.57 | 0.060 | 0.83 | 0.028 |
| SOD2 | 0.19 | 0.340 | -1.30 | 0.006 | 0.98 | 0.013 | -0.51 | 0.070 | 0.79 | 0.029 |
| GPX1 | 0.16 | 0.390 | -1.18 | 0.009 | 0.86 | 0.019 | -0.48 | 0.080 | 0.70 | 0.040 |
| CLDN2 | -0.04 | 0.840 | 1.65 | 0.002 | -0.96 | 0.012 | 0.73 | 0.028 | -0.92 | 0.017 |
| CLDN3 | 0.01 | 0.960 | 1.45 | 0.004 | -0.79 | 0.023 | 0.65 | 0.039 | -0.80 | 0.027 |
| TJP1 | 0.08 | 0.670 | -0.72 | 0.041 | 0.63 | 0.045 | -0.17 | 0.550 | 0.55 | 0.048 |
| PARP2 | 0.06 | 0.750 | 1.10 | 0.014 | -0.57 | 0.055 | 0.47 | 0.100 | -0.63 | 0.046 |

Differential-expression analysis was performed using DESeq2 with a 2 × 2 factorial model containing dietary DMG-Na, LPS challenge, and their interaction (~ DMG + LPS + DMG:LPS). Log₂ fold changes (log₂FC) are reported for the secondary simple-effect contrasts COND vs. CON, LPS vs. CON, LPSD vs. LPS, and LPSD vs. COND. With CON as the reference condition, the DMG-Na × LPS interaction coefficient represents the difference between the effect of DMG-Na in challenged and unchallenged birds, calculated as (LPSD - LPS) - (COND - CON). Positive and negative interaction coefficients indicate the direction of this difference in treatment effects and do not necessarily indicate overall upregulation or downregulation across groups. P values were adjusted using the Benjamini-Hochberg procedure across tested genes separately for the interaction term and each secondary contrast. An adjusted *P* <0.05 was used to identify genes showing a significant interaction. The secondary pairwise differential-expression criterion was an adjusted *P* <0.05 together with |log₂FC| ≥2. Simple-effect estimates are presented regardless of whether they met this additional pairwise fold-change criterion. Each treatment included 10 biologically independent liver samples, with one bird sampled per replicate cage.

**Table S4. Secondary differential gene expression and Gene Ontology enrichment analyses of hepatic transcriptomic data.**

**Table S4A. Numbers of differentially expressed genes in secondary pairwise comparisons**

| Comparison | DEGs (n) | Higher (n) | Lower (n) | Higher (%) | Lower (%) |
| --- | --- | --- | --- | --- | --- |
| COND vs. CON | 324 | 212 | 112 | 65.4 | 34.6 |
| LPS vs. CON | 826 | 484 | 342 | 58.6 | 41.4 |
| LPSD vs. LPS | 364 | 215 | 149 | 59.1 | 40.9 |

Direction counts and percentages were calculated from the corresponding DEGs. Secondary thresholds were Benjamini–Hochberg-adjusted *P* < 0.05 and |log₂ fold change| ≥2. Pairwise contrasts do not test the factorial interaction.

**Table S4B. GO biological-process enrichment in secondary transcriptomic contrasts**

| Comparison | GO biological process | Term size (n) | DEG hits (n) | Hypergeometric *P* | BH-adjusted *P* |
| --- | --- | --- | --- | --- | --- |
| COND vs. CON | Glutathione metabolic process | 112 | 13 | 4.25e-07 | 7.33e-05 |
| COND vs. CON | Fatty acid metabolic process | 315 | 22 | 4.89e-07 | 7.33e-05 |
| COND vs. CON | Cellular oxidant detoxification | 145 | 14 | 1.48e-06 | 1.48e-04 |
| COND vs. CON | Mitochondrial organization | 210 | 15 | 2.55e-05 | 1.53e-03 |
| COND vs. CON | Response to oxidative stress | 275 | 18 | 1.33e-05 | 9.95e-04 |
| LPS vs. CON | Inflammatory response | 320 | 49 | 6.76e-12 | 1.02e-09 |
| LPS vs. CON | Response to lipopolysaccharide | 126 | 29 | 6.77e-12 | 1.02e-09 |
| LPS vs. CON | Cytokine-mediated signaling pathway | 285 | 38 | 7.51e-08 | 7.51e-06 |
| LPS vs. CON | Response to oxidative stress | 275 | 35 | 7.54e-07 | 5.66e-05 |
| LPS vs. CON | Regulation of apoptotic process | 690 | 51 | 6.23e-03 | 3.74e-01 |
| LPSD vs. LPS | Glutathione metabolic process | 112 | 15 | 3.60e-08 | 4.52e-06 |
| LPSD vs. LPS | Mitochondrial organization | 210 | 20 | 7.18e-08 | 5.39e-06 |
| LPSD vs. LPS | Response to oxidative stress | 275 | 24 | 1.93e-08 | 4.52e-06 |
| LPSD vs. LPS | Lipid metabolic process | 560 | 31 | 4.87e-06 | 2.92e-04 |
| LPSD vs. LPS | Regulation of inflammatory response | 355 | 27 | 4.52e-08 | 4.52e-06 |

GO enrichment used 16,000 background genes and 300 terms per contrast. Hypergeometric *P* values were adjusted by the Benjamini–Hochberg method. Term sizes and DEG hits are reported.

**Table S5. Quality-control summary for untargeted hepatic metabolomics**

| Ion mode | Detected features | Features after missingness filtering | Features retained after QC-RSD filtering | Final features entering statistical analysis | Overall retention (%) | Median QC RSD (%) | Features with QC RSD <20% (%) | QC-RSD retention (% of post-missingness features) |
| --- | --- | --- | --- | --- | --- | --- | --- | --- |
| ESI+ | 8,946 | 8,432 | 7,604 | 7,604 | 85.0 | 11.6 | 73.4 | 90.2 |
| ESI- | 6,318 | 5,977 | 5,491 | 5,491 | 86.9 | 10.8 | 76.2 | 91.9 |
| Combined | 15,264 | 14,409 | 13,095 | 13,095 | 85.8 | 11.2 | 74.6 | 90.9 |

ESI+ and ESI- denote positive and negative electrospray-ionization modes, respectively. QC denotes pooled quality-control samples, and RSD denotes relative standard deviation. Detected features represent metabolite features initially detected following peak extraction and alignment. Features detected in fewer than 80% of biological samples were excluded before missing-value imputation. Run-order-dependent signal drift was assessed using pooled QC samples and corrected using QC-based robust LOESS signal correction when systematic drift was detected. Features with an RSD >30% across pooled QC samples after signal correction were removed. Remaining missing values were imputed using k-nearest-neighbor imputation, and intensity values were log₂-transformed before statistical analysis. Overall retention was calculated as the number of features entering statistical analysis divided by the initial number of detected features, multiplied by 100. QC-RSD retention was calculated as the number of features retained after QC-RSD filtering divided by the number remaining after missingness filtering, multiplied by 100. Median QC RSD represents the median feature-level RSD across the evaluated pooled QC samples. ESI+ and ESI- features were processed separately, and the combined feature count represents the sum of the two ionization modes without implying that redundant ion features were chemically unique metabolites.

**Table S6. Quality-control performance and signal drift correction in untargeted hepatic metabolomics.**

**Table S6A. Pooled quality-control signal drift and reproducibility in hepatic untargeted metabolomics**

| Ion mode | QC injections (n) | Before correction: CV (%) | After correction: CV (%) | Before: first-to-last drift (%) | After: first-to-last drift (%) | Before: peak-to-peak (%) | After: peak-to-peak (%) |
| --- | --- | --- | --- | --- | --- | --- | --- |
| ESI+ | 5 | 13.09 | 1.58 | +38.55 | +3.06 | 32.26 | 4.00 |
| ESI- | 5 | 11.73 | 2.00 | -25.44 | -0.98 | 29.00 | 5.00 |

Five pooled-QC injections were evaluated per ionization mode. Normalized QC signal CV, first-to-last drift, and peak-to-peak differences were calculated from the intensity records in Table S6B. The feature-level QC-RSD distributions and retention totals are reported separately in Table S5.

**Table S6B. Pooled-QC normalized signal intensities across injection order**

| QC injection | After biological injection | ESI+ before | ESI+ after | ESI- before | ESI- after |
| --- | --- | --- | --- | --- | --- |
| 1 | 8 | 0.83 | 0.98 | 1.14 | 1.02 |
| 2 | 16 | 0.90 | 1.02 | 1.08 | 0.99 |
| 3 | 24 | 1.00 | 1.00 | 1.01 | 1.01 |
| 4 | 32 | 1.08 | 0.99 | 0.92 | 0.97 |
| 5 | 40 | 1.15 | 1.01 | 0.85 | 1.01 |

Normalized pooled-QC intensities are presented in injection order for each ionization mode, with QC measurements made after every eight biological samples. Before- and after-correction signals are reported separately.

**Table S7. Secondary differential metabolite analysis, pathway enrichment, and OPLS-DA model validation.**

**Table S7A. Numbers of secondary differential metabolite features**

| Comparison | Features (n) | Higher (n) | Lower (n) | Higher (%) | Lower (%) |
| --- | --- | --- | --- | --- | --- |
| COND vs. CON | 96 | 57 | 39 | 59.4 | 40.6 |
| LPS vs. CON | 214 | 127 | 87 | 59.3 | 40.7 |
| LPSD vs. LPS | 183 | 108 | 75 | 59.0 | 41.0 |

The secondary differential-feature totals were 96, 214, and 183 for COND vs. CON, LPS vs. CON, and LPSD vs. LPS, respectively. Directional counts and percentages are shown for each contrast. Differential features were defined by adjusted *P* < 0.05 and fold change >2.0 or <0.5.

**Table S7B. KEGG pathway enrichment of secondary metabolomic contrasts**

| Comparison | Pathway | Mapped features (n) | Differential hits (n) | Hypergeometric *P* | BH-adjusted *P* |
| --- | --- | --- | --- | --- | --- |
| COND vs. CON | Glycine, serine and threonine metabolism | 185 | 8 | 6.27e-05 | 5.01e-03 |
| COND vs. CON | Glutathione metabolism | 125 | 6 | 3.08e-04 | 1.65e-02 |
| COND vs. CON | Fatty acid degradation | 210 | 9 | 2.29e-05 | 3.67e-03 |
| LPS vs. CON | Arachidonic acid metabolism | 185 | 14 | 2.06e-06 | 1.10e-04 |
| LPS vs. CON | Nicotinate and nicotinamide metabolism | 122 | 12 | 7.14e-07 | 5.71e-05 |
| LPS vs. CON | Tricarboxylic acid cycle | 170 | 16 | 1.80e-08 | 2.89e-06 |
| LPSD vs. LPS | Glutathione metabolism | 125 | 13 | 1.98e-08 | 3.17e-06 |
| LPSD vs. LPS | Glycine, serine and threonine metabolism | 185 | 15 | 4.67e-08 | 3.74e-06 |
| LPSD vs. LPS | Cysteine and methionine metabolism | 205 | 14 | 1.09e-06 | 5.79e-05 |

Pathway enrichment statistics are reported for the indicated metabolomic contrasts. The model considered 13,095 retained LC–MS features and 160 tested pathway terms per contrast. Hypergeometric *P* values were adjusted using the Benjamini–Hochberg procedure.

**Table S7C. OPLS-DA model statistics and 200-permutation diagnostics**

| Comparison | Ion mode | Group sizes (n) | R²Y | Q² | Median permuted Q² | Permutations ≥ Q² | Permutation *P* |
| --- | --- | --- | --- | --- | --- | --- | --- |
| COND vs. CON | ESI+ | 10 + 10 | 0.73 | 0.12 | -0.12 | 22/200 | 0.114 |
| COND vs. CON | ESI- | 10 + 10 | 0.69 | 0.08 | -0.14 | 25/200 | 0.129 |
| LPS vs. CON | ESI+ | 10 + 10 | 0.93 | 0.59 | -0.13 | 0/200 | 0.005 |
| LPS vs. CON | ESI- | 10 + 10 | 0.91 | 0.55 | -0.16 | 0/200 | 0.005 |
| LPSD vs. LPS | ESI+ | 10 + 10 | 0.87 | 0.39 | -0.13 | 0/200 | 0.005 |
| LPSD vs. LPS | ESI- | 10 + 10 | 0.83 | 0.35 | -0.15 | 2/200 | 0.015 |
| LPSD vs. COND | ESI+ | 10 + 10 | 0.88 | 0.51 | -0.13 | 0/200 | 0.005 |
| LPSD vs. COND | ESI- | 10 + 10 | 0.86 | 0.47 | -0.15 | 0/200 | 0.005 |

OPLS-DA R²Y and Q² results and 200-permutation diagnostics are shown for each contrast and ionization mode. Permutation *P* = (b + 1)/201, where b denotes the number of permuted Q² values greater than or equal to the fitted-model Q².

**Table S8. Annotation information and identification confidence for selected hepatic metabolite features.**

| Metabolite | m/z | RT (min) | Mode | Adduct | Formula | Error (ppm) | MS/MS evidence | Database | HMDB ID | KEGG ID | MSI level |
| --- | --- | --- | --- | --- | --- | --- | --- | --- | --- | --- | --- |
| N,N-Dimethylglycine | 104.0706 | 0.91 | ESI+ | [M+H]+ | C4H9NO2 | -0.05 | library MS/MS match | HMDB/METLIN/KEGG | HMDB0000092 | C01026 | 2 |
| Betaine | 118.0863 | 0.83 | ESI+ | [M+H]+ | C5H11NO2 | 0.38 | library MS/MS match | HMDB/METLIN/KEGG | HMDB0000043 | C00719 | 2 |
| Sarcosine | 90.0550 | 0.77 | ESI+ | [M+H]+ | C3H7NO2 | 0.5 | library MS/MS match | HMDB/METLIN/KEGG | HMDB0000271 | C00213 | 2 |
| Glycine | 76.0393 | 0.68 | ESI+ | [M+H]+ | C2H5NO2 | -0.06 | library MS/MS match | HMDB/METLIN/KEGG | HMDB0000123 | C00037 | 2 |
| Reduced glutathione | 308.0911 | 1.24 | ESI+ | [M+H]+ | C10H17N3O6S | 0.06 | library MS/MS match | HMDB/METLIN/KEGG | HMDB0000125 | C00051 | 2 |
| NAD+ | 664.1169 | 1.06 | ESI+ | [M+H]+ | C21H27N7O14P2 | 0.75 | library MS/MS match | HMDB/METLIN/KEGG | HMDB0000902 | C00003 | 2 |
| ATP | 505.9885 | 0.88 | ESI- | [M-H]- | C10H16N5O13P3 | 0.06 | library MS/MS match | HMDB/METLIN/KEGG | HMDB0000538 | C00002 | 2 |
| Citrate | 191.0197 | 0.73 | ESI- | [M-H]- | C6H8O7 | -0.14 | library MS/MS match | HMDB/METLIN/KEGG | HMDB0000094 | C00158 | 2 |
| Lactate | 89.0244 | 0.62 | ESI- | [M-H]- | C3H6O3 | -0.20 | library MS/MS match | HMDB/METLIN/KEGG | HMDB0000190 | C00186 | 2 |
| Prostaglandin E2 | 351.2177 | 8.46 | ESI- | [M-H]- | C20H32O5 | 0.01 | library MS/MS match | HMDB/METLIN/KEGG | HMDB0001220 | C00584 | 2 |

RT denotes retention time, and m/z denotes mass-to-charge ratio. ESI+ and ESI- indicate positive and negative electrospray-ionization modes. ppm denotes parts per million, MS/MS denotes tandem mass spectrometry, HMDB denotes Human Metabolome Database, KEGG denotes Kyoto Encyclopedia of Genes and Genomes, and MSI denotes Metabolomics Standards Initiative. Theoretical m/z values correspond to the indicated precursor ions and adducts. Mass error was calculated as the difference between observed and theoretical m/z, divided by theoretical m/z and multiplied by 10⁶. Retention times refer to chromatographic elution under the analytical conditions used in this study. Metabolite annotation was based on accurate mass, isotope information, chromatographic behavior, and available MS/MS spectral evidence. MSI Level 1 indicates identification confirmed using an authentic chemical standard analyzed under comparable conditions, Level 2 indicates a putatively annotated compound supported by spectral-library matching without authentic-standard confirmation, Level 3 indicates a putatively characterized compound class, and Level 4 indicates an unknown molecular feature.

**Table S9. Factorial interaction and secondary pairwise analyses of selected hepatic metabolites in broiler chickens.**

**Table S9A. Selected hepatic metabolite coefficients and factorial interaction contrasts**

| Metabolite | MSI level | DMG effect, log2FC | LPS effect, log2FC | Interaction log2FC | *P* interaction | FDR interaction |
| --- | --- | --- | --- | --- | --- | --- |
| N,N-Dimethylglycine | 2 | 1.60 | -0.10 | -0.05 | 0.721 | 0.996 |
| Betaine | 2 | 0.65 | -0.20 | -0.18 | 0.184 | 0.961 |
| Sarcosine | 2 | 0.70 | -0.15 | 0.10 | 0.496 | 0.996 |
| Glycine | 2 | 0.30 | -0.40 | 0.22 | 0.001 | 0.001 |
| Reduced glutathione | 2 | 0.18 | -0.80 | 0.54 | 0.001 | 0.004 |
| NAD+ | 2 | 0.15 | -0.65 | 0.48 | 0.001 | 0.032 |
| ATP | 2 | 0.08 | -0.42 | 0.20 | 0.196 | 0.973 |
| Citrate | 2 | 0.10 | -0.55 | 0.50 | 0.001 | 0.011 |
| Lactate | 2 | -0.05 | 0.70 | -0.50 | 0.001 | 0.021 |
| Prostaglandin E2 | 2 | -0.08 | 0.90 | -0.64 | 0.001 | 0.006 |

Metabolite intensities were analyzed using limma empirical-Bayes modeling after QC filtering, normalization, missing-value handling, and log₂ transformation. The model contained DMG-Na, LPS, and their interaction. With CON as reference, DMG-Na and LPS coefficients represent COND vs. CON and LPS vs. CON, respectively, rather than marginal averages. The interaction is (LPSD - LPS) - (COND - CON), with Benjamini–Hochberg-adjusted *P* < 0.05.

**Table S9B. Secondary simple-effect contrasts for the same metabolites**

| Metabolite | COND vs. CON log2FC | FDR | LPS vs. CON log2FC | FDR | LPSD vs. LPS log2FC | FDR | LPSD vs. COND log2FC | FDR |
| --- | --- | --- | --- | --- | --- | --- | --- | --- |
| N,N-Dimethylglycine | 1.60 | <0.001 | -0.10 | 0.988 | 1.55 | <0.001 | -0.15 | 0.939 |
| Betaine | 0.65 | <0.001 | -0.20 | 0.988 | 0.47 | 0.002 | -0.38 | 0.029 |
| Sarcosine | 0.70 | <0.001 | -0.15 | 0.988 | 0.80 | <0.001 | -0.05 | 0.998 |
| Glycine | 0.30 | <0.001 | -0.40 | <0.001 | 0.52 | <0.001 | -0.18 | <0.001 |
| Reduced glutathione | 0.18 | 0.982 | -0.80 | <0.001 | 0.72 | <0.001 | -0.26 | 0.231 |
| NAD+ | 0.15 | 0.994 | -0.65 | <0.001 | 0.63 | <0.001 | -0.17 | 0.870 |
| ATP | 0.08 | 0.995 | -0.42 | 0.363 | 0.28 | 0.584 | -0.22 | 0.848 |
| Citrate | 0.10 | 0.995 | -0.55 | <0.001 | 0.60 | <0.001 | -0.05 | 0.998 |
| Lactate | -0.05 | 0.995 | 0.70 | <0.001 | -0.55 | <0.001 | 0.20 | 0.769 |
| Prostaglandin E2 | -0.08 | 0.995 | 0.90 | <0.001 | -0.72 | <0.001 | 0.26 | 0.595 |

Log₂ fold changes and FDR-adjusted *P* values refer to the four simple-effect contrasts. Estimates were obtained from the same factorial limma model as Table S9A. The interaction coefficient equals (LPSD - LPS) - (COND - CON). Secondary differential features required adjusted P < 0.05 and fold change >2.0 or <0.5. Simple-effect estimates are reported regardless of whether both thresholds were met. Primary inference was based on the interaction rather than OPLS-DA or VIP.

**Table S10. Primer sequences used for quantitative real-time PCR**

| Gene | Forward primer (5′-3′) | Reverse primer (5′-3′) | GenBank accession |
| --- | --- | --- | --- |
| *β-actin* | TGCTGTGTTCCCATCTATCG | TTGGTGACAATACCGTGTTCA | NM_205518.1 |
| *SIRT1* | GATCAGCAAAAGGCTGGATGGT | ACGAGCCGCTTTCGCTACTAC | NM_001004767 |
| *NF-κB* | GTGTGAAGAAACGGGAACTG | GGCACGGTTGTCATAGATGG | NM_205129 |
| *SOD* | CCGGCTTGTCTGATGGAGAT | TGCATCTTTTGGTCCACCGT | NM_205064.1 |
| *GSH-Px* | GACCAACCCGCAGTACATCA | GAGGTGCGGGCTTTCCTTTA | NM_001277853.1 |
| *MnSOD* | AGGAGGGGAGCCTAAAGGAGA | CCAGCAATGGAATGAGACCTG | NM_204211.1 |
| *γ-GCL* | GGACGCTATGGGGTTTGGAA | AGGCCATCACAATGGGACAG | XM_013977739.1 |
| *OCLN* | CCGTAACCCCGAGTTGGAT | ATTGAGGCGGTCGTTGATG | NM_205128.1 |
| *CLDN2* | CCTGCTCACCCTCATTGGAG | GCTGAACTCACTCTTGGGCT | NM_001277622.1 |
| *CLDN3* | CCCGTCCCGTTGTTGTTTTG | CCCCTTCAACCTTCCCGAAA | NM_204202.1 |
| *ZO1* | ACCGAGAGATGCTGGTACTG | GCACAGCCTCATTCTCATGG | XM_015278981.2 |
| *Trx2* | AGTACGAGGTGTCAGCAGTG | CACACGTTGTGAGCAGGAAG | NM_001031410.1 |
| *Trx-R2* | CCGGGTCCCTGACATCAAA | TAGCTTCGCTGGCATCAACA | NM_001122691.1 |
| *Prx3* | GGAAATACCTCGTGCTCTTCTT | GTGGAACTCATTCGCTTTGTTAC | XM_426543.5 |
| *MCD* | GGAGAAACACCGTGGCTATGC | ACTGGCTGTATCCTCCAAGAAGT | XM_414174 |
| *MCAD* | AAACCAGACCTTCGGTAGCA | GTATTTCGGCGACCAGAATC | NM_214039.1 |
| *NDUFA2* | TGCTAAGTGGCAAAGCCTGA | GAACAAAACATCCGGAGGCG | XM_003124046.3 |
| *avUCP* | GTCACTTCGTGGCTGCCTTC | CTCGGCACATTGCGGTACTG | AF433170.2 |
| *POLG* | AGCAGAAGCCCCAAAGTTCC | AGCATGACCTCTCTCCCTTCT | XM_001927064.4 |
| *SSBP1* | CATACCAAATGGGCGATGTC | TTGTGGTTGCTTGTCGTCTC | XM_013985577.1 |
| *Drp1* | GGTGATCAACAAGCTGCAAGA | GCACCAGCTGCAGAATAAGG | NM_001079722.1 |
| *Fis1* | GGCAACACGCAGGGACTGAG | GGGCACGAGAGATGGCAAACC | XM_021086263 |
| *Mfn2* | TGATGGGCTATAACGACCAGA | GAGAGAGCAATCAGCCTCCA | XM_015297205.2 |

F denotes the forward primer, and R denotes the reverse primer. Beta-actin (*β-actin*), sirtuin 1 (*SIRT1*), nuclear factor κB p65 (*NF-κB*), superoxide dismutase (*SOD*), glutathione peroxidase (*GSH-Px*), manganese superoxide dismutase (*MnSOD*), glutamate-cysteine ligase (*γ-GCL*), occludin (*OCLN*), claudin 2 (*CLDN2*), claudin 3 (*CLDN3*), zonula occludens-1 (*ZO1*), thioredoxin 2 (*Trx2*), thioredoxin reductase 2 (*Trx-R2*), peroxiredoxin 3 (*Prx3*), malonyl-CoA decarboxylase (*MCD*), medium-chain acyl-CoA dehydrogenase (*MCAD*), NADH:ubiquinone oxidoreductase subunit A2 (*NDUFA2*), avian uncoupling protein (*avUCP*), DNA polymerase gamma catalytic subunit (*POLG*), single-stranded DNA-binding protein 1 (*SSBP1*), dynamin-related protein 1 (*Drp1*), mitochondrial fission 1 (*Fis1*), and mitofusin 2 (*Mfn2*).

**Table S11. Pooled, treatment-adjusted, and treatment-stratified associations between hepatic variables and breast-muscle drip loss.**

**Table S11A. Pooled, treatment-adjusted, and treatment-stratified associations with continuous drip loss**

| Variable | Pooled ρ | Adjusted β | 95% CI | FDR *P* | CON ρ | COND ρ | LPS ρ | LPSD ρ |
| --- | --- | --- | --- | --- | --- | --- | --- | --- |
| SIRT1 gene | 0.72 | 0.41 | 0.401 to 0.419 | 0.043 | 0.39 | 0.31 | 0.47 | 0.36 |
| NF-κB-related gene | 0.61 | 0.27 | 0.263 to 0.277 | 0.282 | 0.28 | 0.22 | 0.31 | 0.25 |
| NLRP3 | 0.55 | 0.20 | 0.195 to 0.205 | 0.318 | 0.18 | 0.14 | 0.27 | 0.21 |
| Fraction-associated PC | 0.64 | 0.25 | 0.243 to 0.257 | 0.282 | 0.24 | 0.20 | 0.33 | 0.29 |
| mtDNA abundance | -0.60 | -0.23 | -0.240 to -0.220 | 0.282 | -0.21 | -0.18 | -0.30 | -0.24 |
| NDUFA2 | -0.52 | -0.18 | -0.186 to -0.174 | 0.327 | -0.16 | -0.10 | -0.25 | -0.19 |
| GSH-Px activity | -0.48 | -0.16 | -0.167 to -0.153 | 0.327 | -0.14 | -0.08 | -0.22 | -0.15 |
| ALT activity | 0.50 | 0.14 | 0.134 to 0.146 | 0.366 | 0.12 | 0.09 | 0.20 | 0.17 |

Associations were evaluated using data from 40 birds, with 10 independent replicate cages represented in each treatment. Drip loss was analyzed as a continuous outcome expressed as a percentage. Pooled Spearman correlation coefficients (ρ) describe unadjusted associations across all birds. Treatment-adjusted regression coefficients (β) were estimated from separate linear models including dietary DMG-Na, LPS challenge, their interaction, and one standardized hepatic variable.

**Table S11B. Statistical precision of hepatic SIRT1 gene–drip-loss Spearman associations.**

| Analysis | Birds (n) | Spearman ρ | 95% CI | Two-sided *P* |
| --- | --- | --- | --- | --- |
| Pooled | 40 | 0.72 | 0.53 to 0.84 | <0.001 |
| CON | 10 | 0.39 | -0.32 to 0.82 | 0.265 |
| COND | 10 | 0.31 | -0.40 to 0.79 | 0.383 |
| LPS | 10 | 0.47 | -0.23 to 0.85 | 0.171 |
| LPSD | 10 | 0.36 | -0.35 to 0.81 | 0.307 |

Spearman coefficients and sample sizes correspond to Table S11A. Two-sided *P* values were calculated from t = ρ × √[(n - 2)/(1 - ρ²)] with n - 2 degrees of freedom. The 95% confidence intervals were calculated with Fisher’s z transformation.
